# Linking resource selection to population performance spatially to identify species' habitat across broad scales: An example of greater sage‐grouse in a distinct population segment

**DOI:** 10.1002/ece3.10891

**Published:** 2024-10-10

**Authors:** Megan C. Milligan, Peter S. Coates, Brianne E. Brussee, Shawn T. O'Neil, Steven R. Mathews, Shawn P. Espinosa, Katherine Miller, Daniel Skalos, Lief A. Wiechman, Steve Abele, John Boone, Kristie Boatner, Heather Stone, Michael L. Casazza

**Affiliations:** ^1^ U.S. Geological Survey Western Ecological Research Center Dixon California USA; ^2^ Nevada Department of Wildlife Reno Nevada USA; ^3^ California Department of Fish and Wildlife Sacramento California USA; ^4^ U.S. Geological Survey Fort Collins Science Center Fort Collins Colorado USA; ^5^ U.S. Fish and Wildlife Service Reno Nevada USA; ^6^ Great Basin Bird Observatory Reno Nevada USA; ^7^ U.S. Forest Service Sparks Nevada USA; ^8^ Bureau of Land Management Bishop California USA

**Keywords:** demographic response, greater sage‐grouse, habitat selection, life stages, mapping, reproduction, sagebrush ecosystem, survival

## Abstract

Management decisions often focus on the habitat selection of marked individuals without considering the contribution to demographic performance in selected habitats. Because habitat selection is not always adaptive, understanding the spatial relationship between habitat selection and demographic performance is critical to management decisions. Mapping both habitat selection and demographic performance for species of conservation concern can help guide population‐scale conservation efforts. We demonstrate a quantitative approach to differentiate areas supporting selection and survival at large spatial extents. As a case study, we applied this approach to greater sage‐grouse (*Centrocercus urophasianus*; hereafter, sage‐grouse), an indicator species for sagebrush ecosystems. We evaluated both habitat selection and survival across multiple reproductive life stages (nesting, brood‐rearing) in the Bi‐State Distinct Population Segment, a genetically distinct and geographically isolated population of sage‐grouse on the southwestern edge of the species' range. Our approach allowed us to identify both mismatches between selection and survival and trade‐offs between reproductive life stages. These findings suggest resource demands vary across time, with predation risk being a dominant driver of habitat selection during nesting and early brood‐rearing periods when chicks are smaller and flightless, whereas access to forage resources becomes more important during late brood rearing when resources become increasingly limited. Moving beyond identifying and managing habitat solely based on species occupancy or use by incorporating demographic measures allows managers to tailor actions to their specific goals; for example, protections of areas that support high selection and high survival and restoration actions focused on increasing survival in areas of high selection and low survival.

## Graphical abstract

Habitat selection is not always adaptive, so understanding the link between habitat selection and demographic performance is critical to management decisions. We evaluated both selection and survival across multiple reproductive life stages in a genetically distinct and geographically isolated population of greater sage‐grouse, an indicator species for sagebrush ecosystems. Our approach allowed us to identify both mismatches between selection and survival and trade‐offs between life stages.

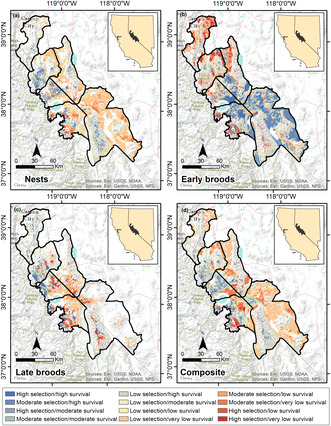

## 1 INTRODUCTION

With habitat loss and degradation representing one of the greatest threats to wildlife (Caro et al., 2022), identifying high‐quality habitats is critical to meeting specific management objectives. Animal distribution or occupancy have been widely adopted as proxies for habitat quality, under the assumption that individuals will occupy higher quality habitat given a choice (Fretwell & Lucas, 1970; Stephens et al., 2015). While they represent indirect assessments of habitat quality, measures of distribution or occupancy have gained traction because they are relatively easy and inexpensive to obtain. In contrast, direct measures, such as reproduction or survival, often necessitate longer studies and can require individually marked animals (Heinrichs et al., 2010; Johnson, 2007; Jones, 2001). However, if habitat conditions supporting higher densities do not favor reproduction and survival, this could lead to the prioritization of lower quality habitats for conservation (Heinrichs et al., 2010; Van Horne, 1983). Thus, understanding the spatial relationship between habitat selection and demographic performance is critical for effective species conservation (Heinrichs et al., 2010; Matthiopoulos et al., 2015).

Habitat selection is often assumed to be an adaptive behavior, with individuals selecting specific features to maximize their fitness (Fretwell & Lucas, 1970), but selection can become nonideal or even maladaptive when individuals select areas that compromise their survival or reproductive success (Battin, 2004). As humans alter the environment rapidly and at large spatial extents (Millennium Ecosystem Assessment, 2005), specialist species and those with rigid behaviors cannot adapt to the changing environment quickly. As a result, they may make poor habitat selection decisions due to a disconnect between cues that were successful previously but may not be indicative of current habitat quality (Merkle et al., 2022). Such disconnects could occur for a variety of reasons, including an inability to correctly assess habitat quality or trade‐offs where selection that maximizes one aspect of fitness has a negative effect on another aspect (Battin, 2004; Stamps et al., 2005). For example, increased frequency and extent of wildfires in the western United States, in part due to the positive feedback loop between fire and invasive annual grasses, has created a population sink for greater sage‐grouse (*Centrocercus urophasianus*; hereafter, sage‐grouse), where strong fidelity led birds to nest in areas where they experienced lower reproductive success (O'Neil et al., 2020). Identifying where habitat selection is adaptive therefore requires evaluating both selection and demographic performance across multiple life stages using covariates that vary both spatially and temporally to gain a complete picture of habitat requirements and constraints (Johnson, 2007).

Evaluating the spatial distribution of resources linked to both habitat selection and demographic performance can highlight areas selected by animals that improve their survival and identify areas with misalignment between selection and survival to inform restoration or habitat improvement actions (Crawford et al., 2020; Saher et al., 2022). This is particularly critical in cases of maladaptive habitat selection, where individuals are selecting areas despite experiencing poor demographic performance (Pulliam, 1988). Identifying appropriate management actions requires distinguishing between productive habitat (i.e., increased selection and survival) and ecological traps, where an individual preferentially selects habitat that reduces its fitness (Kristan III, 2003; Robertson & Hutto, 2006). In a metapopulation, ecological traps are a form of habitat sinks, affecting population dynamics and persistence at large spatial scales and, if abundant enough, forcing populations toward extirpation (Hanski & Gilpin, 1991; Howe et al., 1991). While quantitatively differentiating productive areas from habitat sinks can be challenging, it can provide valuable information for management. For example, conservation actions aimed at modifying the environment to increase survival in areas selected by animals can “switch” a habitat sink to a productive habitat and thereby increase metapopulation persistence. Previous studies linking habitat selection and survival have been restricted to a single life stage or limited spatial extent (Aldridge & Boyce, 2007). However, focusing on a single life stage does not provide a complete picture of species' habitat requirements across their different life history phases.

We evaluated both habitat selection and survival across multiple reproductive life stages to better understand the spatial relationship between these two processes and provide a method for prioritizing habitat by linking resource selection to demographic performance. As a case study, we monitored radio‐marked sage‐grouse from 2003 to 2019 in the Bi‐State Distinct Population Segment (DPS) during both reproductive life stages of nesting and brood‐rearing, which are important determinants of population growth (Taylor et al., 2012) and typically the target of management actions. Sage‐grouse are sagebrush obligate species and central to land management plans across much of the western United States, largely because of their status as an indicator species for the condition of sagebrush ecosystems (Hanser & Knick, 2011), which are considered among the most imperiled ecosystems in North America (Noss et al., 1995). The Bi‐State DPS is a genetically distinct and geographically isolated population on the southwestern edge of the sage‐grouse geographic range whose population trends are disconnected from the closest sage‐grouse populations in the Great Basin (Coates, Ricca, et al., 2020). The DPS has been evaluated for listing under the Endangered Species Act separately from other populations (U.S. Fish and Wildlife Service, 2015). Mapping sage‐grouse habitat suitability during reproductive phases using a selection and survival modeling approach can guide conservation and restoration efforts in threatened sagebrush ecosystems and demonstrate how this approach can be applied to other species.

## 2 METHODS

### 2.1 Study area

Our study focused on the Bi‐State DPS (16,880 km^2^), which spans portions of the border of Nevada and California and is situated in the topographically diverse area between the Sierra Nevada and the Great Basin. Vegetation predominantly consisted of mountain big sagebrush (*Artemisia tridentata vaseyana*), with some areas of low sagebrush (*A*. *arbuscula*), Wyoming big sagebrush (*A*. *t*. *wyomingensis*), silver sagebrush (*A*. *cana*), and basin big sagebrush (*A*. *t*. *tridentata*) occurring locally. Invasive cheatgrass (*Bromus tectorum*) was present but uncommon. Two native conifers, single‐leaf pinyon (*Pinus monophyla*) and Utah juniper (*Juniperus osteosperma*), occurred at higher elevations and in mesic sagebrush environments.

### 2.2 Field methods

Adult and juvenile sage‐grouse were captured across 10 subpopulations within the Bi‐State DPS (Appendix S1) from 2003 to 2019 in the spring, summer, and fall seasons using standard spotlighting techniques and were classified by age and sex using published methodologies (Ammann, 1944). Captured sage‐grouse were fitted with either necklace‐style very high‐frequency radio‐transmitters (Advanced Telemetry Systems, Isanti, Minnesota) or rump‐mounted Global Positioning System platform transmitting terminals (GeoTrack, Apex, North Carolina). All sage‐grouse were captured and handled in accordance with the USGS Western Ecological Research Center (WERC) Animal Care and Use Protocol WERC‐2015‐02.

Radio‐marked birds were located using portable receivers and handheld Yagi antennas during nesting and brood‐rearing periods ≥2 times per week. To identify nest locations, we avoided flushing females and visually confirmed nesting status for females that were relocated in the same locations during two consecutive checks, which indicated that incubation had begun. Nests were considered successful if ≥1 chick hatched, as determined by a visual assessment of eggshell remains or by observing chicks in the nest bowl. Females with successful nests were then located and checked for brood presence every 10 days for up to 50 days, with checks conducted both during daylight and nighttime hours, which allowed the presence of chicks with the marked female to be checked with spotlights. At 50 days posthatch, after which young are capable of surviving independently, the brood was flushed and considered successful if ≥1 chick survived.

### 2.3 Spatial data

We evaluated the effects of multiple spatially and temporally explicit environmental covariates on sage‐grouse habitat selection and survival across reproductive life stages (Table 1). For temporally varying covariates, we used data from the year a nest or brood was monitored. We evaluated the selection of land cover variables at multiple spatial scales because animals often select habitat in a scale‐dependent manner (Boyce, 2006). For all analyses, we calculated the proportion of each land cover covariate or density of feature (e.g., streams) within circular moving windows with radii of 167.9, 439.5, and 1451.7 m, which represented averages of minimum, mean, and maximum daily distances traveled by sage‐grouse (Coates, Casazza, et al., 2016). We included additional scales for nest and brood analyses based on the assumption that movement patterns would differ during these life stages because a female is tied to either a single nest site or a brood with limited mobility. We included a finer scale (radius = 75 m), representing the core recess area used by nesting females, and an intermediate scale (260 m), representing the average distance moved during nesting recesses (Dudko et al., 2018). We included an additional scale that represented the median movement distance of a female with broods within either the early or late brood‐rearing period (early: 260 m; late: 370 m, Coates et al., 2024). For distance‐based predictors, we transformed distances using an exponential decay function, where e=−d/α and α was the mean value at all locations for a given analysis (Coates, Casazza, et al., 2016). This allowed for the effect of a predictor to decay with increasing distance. All variables were scaled and centered prior to analysis.

**TABLE 1:** Variables evaluated in analyses examining habitat selection and survival for greater sage‐grouse nests and broods in the Bi‐State Distinct Population Segment from 2003 to 2019.

Variable	Category	Description/source	References
% Sagebrush	Shrubs	Rangeland Condition Monitoring Assessment and Projection (RCMAP) time‐series layers	Rigge et al. (2021)
Sagebrush height	Shrubs	RCMAP basemap	Rigge et al. (2020)
% Shrubs	Shrubs	RCMAP time‐series	Rigge et al. (2021)
Shrub height	Shrubs	RCMAP basemap	Rigge et al. (2020)
% Herbaceous vegetation	Herbaceous/wet meadows	RCMAP time‐series	Rigge et al. (2021)
% Bare ground	Bare ground	RCMAP time‐series	Rigge et al. (2021)
Annual grass cover^a^	Annual grass	Invasive annual grass cover	Boyte and Wylie (2016)
Pinyon‐juniper cover	Conifer cover	2 cover classes of pinyon‐juniper cover (Phase 1/CC1 = 0%–10% tree canopy cover, Phase 2/CC2 = 10%–20% tree canopy cover)	Gustafson et al. (2018)
Annual burned area^a^	Burned area	Annual burned area with simulated recovery	O'Neil et al. (2020)
Streams	Streams	National Hydrography Dataset	U.S. Geological Survey (2017a)
Springs	Springs	National Hydrography Dataset	U.S. Geological Survey (2017a)
Wet meadows	Herbaceous/wet meadows	USFWS National Wetland Inventory	U.S. Fish and Wildlife Service (2022)
Saline lakes	Saline lakes	National Hydrography Dataset	U.S. Geological Survey (2017a)
Elevation	Elevation	Extracted from digital elevation model (DEM)	U.S. Geological Survey (2017b)
Slope	Topography	Calculated from DEM	Evans and Oakleaf (2012)
Topographic roughness	Topography	Calculated from DEM	Evans and Oakleaf (2012)
Heat load index (HLI)	Temperature/moisture	Calculated from DEM	Evans and Oakleaf (2012)
Compound topographic index (CTI)	Temperature/moisture	Calculated from DEM	Evans and Oakleaf (2012)
Transformed aspect	Temperature/moisture	Calculated from DEM	Evans and Oakleaf (2012)

*Note*: Temporally varying variables were available across the entire study period. The spatial resolution for all variables was 900 m^2^ unless otherwise noted.

^a^ Spatial resolution: 62,500 m^2^.

### 2.4 Analysis

We evaluated both habitat selection and survival for the reproductive life stages of nesting and brood rearing. The latter stage was further divided into early (≤21 days posthatch) and late (>21 days posthatch) brood‐rearing periods (Blomberg et al., 2014). We evaluated habitat selection using resource selection functions (RSFs), comparing used and available points. Our selection model took the form:

$$\text{logit}\left(Y\right)=\beta_{0}+\mathrm{X\beta}+\kappa +\eta +\nu ,$$

where $\beta_{0}$ represents the baseline intercept, Xβ was a vector of selection coefficients β multiplied by fixed habitat covariates X, and κ, η, and ν were random intercepts for site, year, and bird or brood, depending on the analysis. Used points (Y = 1) were from telemetry locations collected from 2003 to 2019. We defined availability based on the movement patterns of monitored sage‐grouse and sampled available points (Y = 0) within the 90th percentile of the distance traveled from leks or an individual's nest for nest and brood selection, respectively. We used the 90th percentile as a cutoff to capture sage‐grouse movement patterns while excluding extreme values that could bias the availability sample. Available points were sampled at a 5:1 available:used ratio to capture heterogeneity in this ecosystem while limiting computer processing time. We also included distance to lek or nest as confounder covariates in the nest and brood analyses, respectively, to account for sage‐grouse movement behavior that might otherwise produce spurious associations with environmental covariates. Importantly, the logistic regression function above was used only to estimate coefficients, whereas the RSF was generated by discarding intercepts and applying the exponential link function (Johnson et al., 2006; McDonald, 2013; Northrup et al., 2013).

For survival, we used binomial models that accounted for exposure time in a Bayesian framework (Shaffer, 2004; Sinnott et al., 2022). Daily survival was calculated as:

$$\text{logit}\left(DS_{i}\right)=\gamma_{0}+\mathrm{X\beta}+\kappa +\eta ,$$

where subscript *i* references individual intervals, κ and η represent random intercepts for site and year, respectively, and daily survival is modeled as a vector of selection coefficients β multiplied by fixed habitat covariates X. The survival probability of a nest or brood *h* over interval *i* was assumed to follow a Bernoulli distribution, $y_{h,i}\sim \text{Bernoulli}\left(\theta_{h,i}\right)$, where $y_{h,i}=1$ if the nest or brood survived the interval, $y_{h,i}=0$, if the nest or brood failed, and $\theta_{h,i}$ is the probability of the nest or brood surviving the interval, which equaled ${\mathrm{DS}}_{h,i}^{t}$ (the daily survival probability (DS) for nest or brood *h* raised to the length (time = *t*) of interval *i*). Encounter histories were structured to represent the interval between successive relocations of a nest or brood and included the day of entry, day of exit, length of the interval, the fate of the nest or brood, and the habitat features measured at the beginning of each interval (Coates et al., 2024).

Relationships between wildlife and habitat are typically scale‐dependent, and rarely does a single “best” scale for all habitat variables exist (Stuber & Fontaine, 2019). Therefore, we first used Bayesian latent indicator variable scale selection to select the most influential scale among each group of variables (BLISS; Stuber et al., 2017). BLISS uses latent scale indicator variables estimated with reversible‐jump Markov chain Monte Carlo (MCMC) sampling to select the best scale within a group without issues of collinearity (Stuber et al., 2017). Variables were grouped into 12 categories to represent similar features and correlated variables (|*r*| ≥ .5), such that no variables between groups were highly correlated (Table 1).

We then developed a final model with only the most influential variables from each group to allow for straightforward inferences. We calculated the probability of direction (*p*(|*β*| > 0); Makowski et al., 2019) and considered probabilities ≥.85 and ≥.95 to represent moderate and strong effects, respectively. For each variable in the final selection models, we calculated relative selection strengths (RSS), which provide an estimate of the relative intensity of two locations differing by one standard deviation, assuming that both locations were equally available (Avgar et al., 2017). An RSS >1 represented a positive effect on habitat selection, whereas an RSS <1 represented a negative effect.

We fit all models using MCMC simulations with JAGS (Plummer, 2003) implemented via the “R2Jags” package (Su & Yajima, 2015) using Program R (R Core Team, 2021). Vague normal priors were used for random effects and their measures of error (*μ* = 0, *σ* ^2^ = Uniform (0, 100); Kéry, 2010). To prevent overfitting, we specified Lasso (i.e., Laplace) prior distributions for each habitat predictor, with an uninformative hyperprior specified for the tuning parameter lambda (Uniform(0.001, 10); Park & Casella, 2008; Hooten & Hobbs, 2015). Each model ran for 30,000 iterations, thinned by a factor of 5, and we discarded the first 20,000 samples and made inferences based on the remaining 10,000 samples from 3 independent MCMC chains. We assessed convergence and MCMC chain mixing visually and based on Gelman‐Rubin convergence statistics (<1.1; Gelman & Hill, 2006).

### 2.5 Model validation

After choosing a final model for each reproductive life stage, we used independent testing data from 50 individual birds or broods randomly withheld to validate the model's ability to predict nest and brood locations. We used cross‐validation methods for RSFs (Johnson et al., 2006) and used‐habitat calibration (UHC) plots (Fieberg et al., 2018) to validate selection models. UHC plots compare observed distributions for each habitat variable to the expected distribution based on the model (Fieberg et al., 2018), using the fitted RSF to generate a predictive distribution of used habitat, which is then compared to the independent testing data for each habitat variable. For survival models, we generated survival predictions based on the posterior distributions from the final model to create predicted survival curves which were then compared to the observed survival curves from the independent testing data, which resulted in a post‐hoc Bayesian predictive *p*‐value (Gelman et al., 2013), where values near 0 or 1 indicate poor model fit (Schmidt et al., 2010).

### 2.6 Selection and survival mapping

Using estimates from the final models and the spatial data layers described above, we mapped both selection and survival for each reproductive life stage across the Bi‐State DPS (Coates et al., 2024). We assumed that the median value of the posterior distribution represented the best estimate for each covariate and applied these in the model equations outlined above, where the matrix **X** represented the relevant raster values for each covariate across each 900 m^2^ pixel in the Bi‐State DPS. For time‐varying covariates, we used the raster values from 2021, which represented the most recent layers available. We did not include distance to lek or nest in the habitat selection maps because we were primarily interested in the spatial distribution of areas suitable for sage‐grouse based on underlying habitat characteristics. Including distance to lek would provide information on the distribution of occupied habitat, which we addressed in a map of example management categories (described below) following similar methods to Coates, Casazza, et al. (2016).

For selection maps, we transformed estimates using a habitat selection index (HSI), where $\mathrm{HSI}=\frac{w\left(x\right)}{1+w\left(x\right)}$, which indicates relative habitat use proportional to availability on a scale of 0–1 (Coates, Casazza, et al., 2016). We then categorized the continuous selection surface into four categories (nonhabitat, low, moderate, and high selection) using the percent isopleth method at used locations with cutoff values at the 50th, 25th, and 5th percentiles (Doherty et al., 2016; O'Neil et al., 2020). To make population‐level inferences on survival, we exponentiated values of daily survival based on the number of days in either the nesting or brood‐rearing periods (nesting = 38 days, early brood‐rearing = 21 days, late brood rearing = 28 days) to calculate cumulative survival for each period. We classified the continuous survival surface for each life stage into four categories (very low, low, moderate, and high survival) based on the distribution of values at failed and successful points following O'Neil et al. (2020).

We then used continuous selection and survival maps for each reproductive life stage to calculate composite selection and survival indices representative of the entire reproductive period. To calculate the composite selection index, we first relativized the habitat selection indices described above by dividing by the maximum value in that life stage (Coates, Brussee, et al., 2020), weighting all three reproductive life stages equally when calculating a composite surface. We then multiplied the three relativized surfaces together to create a composite selection index, which we categorized using the method described above. Calculating the survival index followed a similar procedure, but values were not relativized because the outputs represented true survival probabilities. We multiplied the exponentiated survival surfaces that represented overall survival during a given life stage to create a composite survival index, which we classified following the same methods used for individual survival maps. We only calculated the survival index for pixels that were considered high, moderate, or low based on the composite selection index, thus excluding areas considered nonhabitat.

We then created a ranked index that represented the overlap between the categorized selection and survival maps for each life stage and the composite reproductive indices. The highest habitat rank occurred in pixels that had both high selection and survival and thus were assumed to represent productive habitat for that life stage. In contrast, the lowest rank occurred in pixels with high selection but very low predicted survival, which could indicate maladaptive habitat selection.

Finally, we mapped specific example management categories representing the overlap between the composite selection index and an abundance and space use index (ASUI) calculated from breeding surveys (Coates, Casazza, et al., 2016), which allowed us to highlight high‐quality habitat currently occupied by sage‐grouse. The abundance and space use index represented the distribution of habitat predicted to be occupied by sage‐grouse by accounting for the configuration of leks, the distance to lek, and the predicted abundance at each lek over time. By combining the abundance and space use index with the selection and survival maps described here, we were able to differentiate between likely occupied habitat and potential habitat with low or no occupancy. We defined four example management categories (Coates, Casazza, et al., 2016): (1) priority+ areas, defined as important source habitat for each reproductive life stage supporting high selection and high survival within high‐use areas based on the ASUI, (2) priority areas, defined as areas with either predicted high, moderate, or low selection (excluding nonhabitat) and high use based on the ASUI or source areas outside of high use areas based on the ASUI, (3) general areas, defined as the intersection with high selection and low to no use based on the ASUI or nonhabitat within areas of high use based on the ASUI, and (4) other areas, defined as moderate selection within areas of low to no use based on the ASUI. We provide data to support all models, along with resulting raster surfaces to support land management and conservation efforts, via the USGS *ScienceBase* digital repository (Coates et al., 2024).

## 3 RESULTS

Overall, 579 nests belonging to 419 females were monitored between 2003 and 2019. Average nest survival (95% CRI) over the 38‐day laying and incubation period was 0.38 (0.31–0.47). A total of 285 broods were monitored during the early period (≤21 days posthatch) and 209 during the late period. Survival across the entire 50‐day brood‐rearing period was 0.74 (0.45–0.91).

### 3.1 Nest selection and survival

**FIGURE 1:**
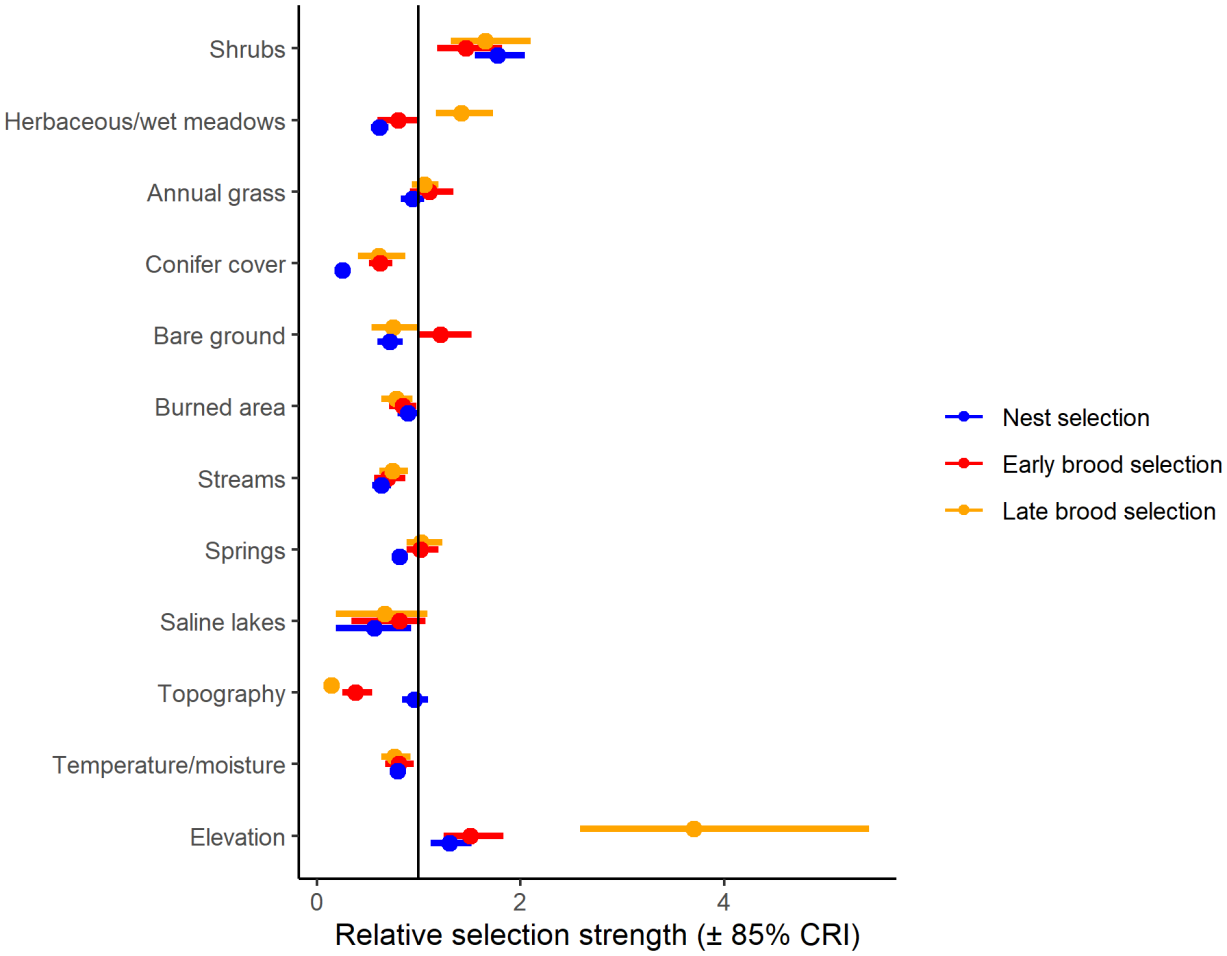
Relative selection strength (± 85% credible intervals) of variables predicting nest (blue), early brood (red), and late brood selection (orange) for greater sage‐grouse in the Bi‐State Distinct Population Segment from 2003 to 2019. Values above one (solid black line) correspond to selection, and values below one correspond to avoidance. For distance variables, estimates below one correspond to a higher selection closer to the linear feature.

For nest sites, the relative probability of selection increased with greater sagebrush height and at higher elevations (Figure 1, Appendix S2). The relative probability of nest site selection decreased in areas with more herbaceous cover, pinyon‐juniper cover, bare ground, burned area, streams, springs, saline lakes, and south‐southwesterly aspects (Figure 1). Our nest selection model had reasonable out‐of‐sample predictive capabilities (Spearman's rank *ρ* = .98, *R* ^2^ = .85, *β*_predict_ = .64) and used‐habitat calibration plots using an independent dataset demonstrated that model‐predicted habitat was consistent with observed nest locations (Appendix S2).

**FIGURE 2:**
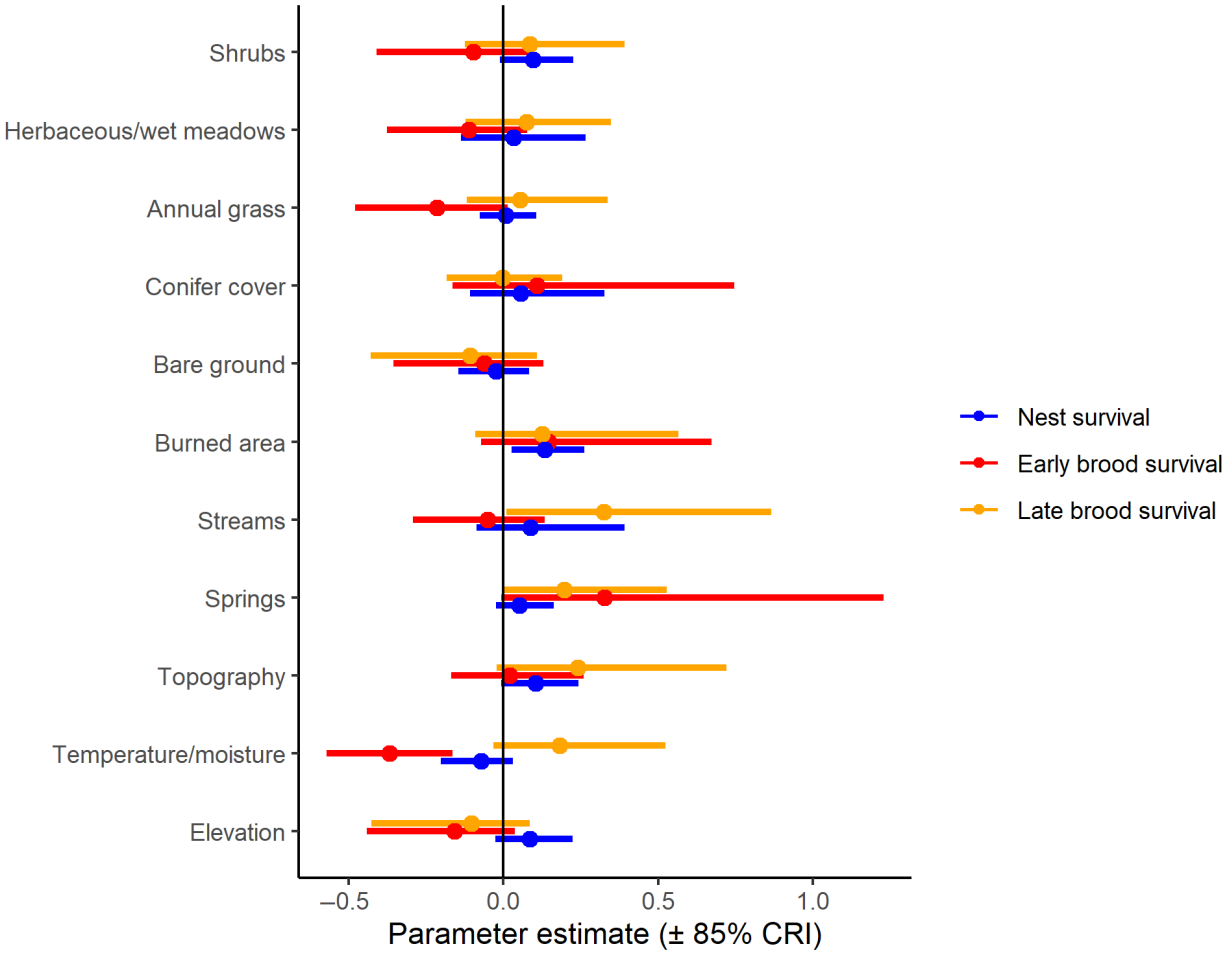
Parameter estimates (± 85% credible intervals) of variables predicting nest (blue), early brood (red), and late brood survival (orange) for greater sage‐grouse in the Bi‐State Distinct Population Segment from 2003 to 2019. Positive estimates for distance variables correspond to higher survival closer to the linear feature.

Nest survival was higher with more burned area, more shrubs, more topographic roughness, and at higher elevations (Figure 2, Appendix S2). Selection and survival were misaligned for burned areas. However, data from within burned areas was limited, with only 2% of nests within a fire perimeter, so we interpreted the moderately positive relationship with caution. The predicted survival curves from the model were consistent with the testing data (Bayesian *p*‐value = .55).

### 3.2 Early brood selection and survival

The relative probability of selection during early brood rearing increased with greater sagebrush height, more bare ground, and at higher elevations (Figure 1, Appendix S2). The relative probability of selection during early brood‐rearing decreased with more wet meadows, pinyon‐juniper cover, more cumulative burned area, more perennial streams, greater topographic roughness, and a higher heat load index (Figure 1). Our early brood selection model had strong out‐of‐sample predictive capabilities (Spearman's rank *ρ* = .99, *R* ^2^ = .96, *β*_predict_ = 1.00), and used‐habitat calibration plots using an independent dataset demonstrated that model‐predicted habitat was consistent with observed early brood locations (Appendix S2).

Early brood survival increased with more burned area and closer to conifer cover (Figure 2, Appendix S2), and decreased with more shrubs and more perennial streams (Figure 2). The predicted survival curves from the model were consistent with the testing data (Bayesian *p*‐value = .55). Selection and survival differed with moderate or strong effects for shrubs, burned area, and conifer cover. Broods selected for areas with taller sagebrush, but survival was higher in areas with lower shrub density. Early broods were more likely to select areas with less burned area closer to conifer cover, which was associated with lower survival, although there was high uncertainty around the effect of conifer cover on early brood survival.

### 3.3 Late brood selection and survival

The relative probability of selection during late brood rearing increased with greater sagebrush height, more herbaceous cover, and at higher elevations (Figure 1, Appendix S2). The relative probability of selection during late brood rearing decreased with greater pinyon‐juniper cover, more bare ground, more cumulative burned area, more perennial streams, more saline lakes, steeper slopes, and more south‐southwesterly aspects (Figure 1). Our late brood selection model had reasonable out‐of‐sample predictive capabilities (Spearman's rank *ρ* = .95, *R* ^2^ = .62, *β*_predict_ = .60), and used‐habitat calibration plots using an independent dataset demonstrated that model‐predicted habitat was consistent with observed late brood locations (Appendix S2).

Late brood survival increased with more intermittent streams, more springs, steeper slopes, and higher values of the compound topographic index (Figure 2, Appendix S2). The predicted survival curves from the model were consistent with the testing data (Bayesian *p*‐value = .55).

Two variables differed across selection and survival with moderate or strong effects: streams and slope. Selection was lower but survival was higher in places with more perennial or intermittent streams, respectively. During late brood‐rearing, selection was higher but survival was lower in areas with steeper slopes.

### 3.4 Mapping

**FIGURE 3:**
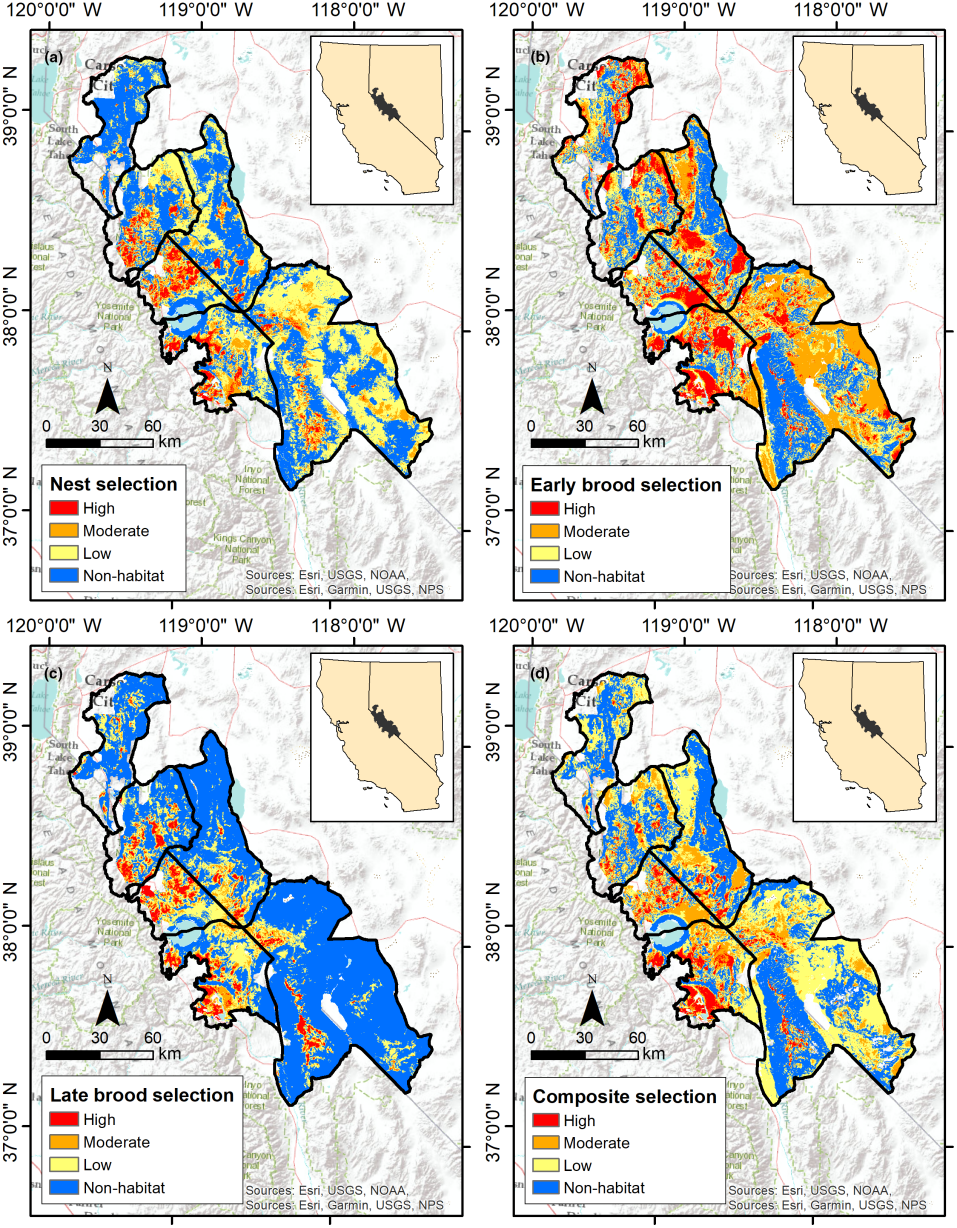
Maps of categorized habitat selection for greater sage‐grouse in the Bi‐State Distinct Population Segment from 2003 to 2019 during nesting (a), early brood‐rearing (b), late brood‐rearing (c), and a composite selection index of the 3 reproductive life stages (d).

**FIGURE 4:**
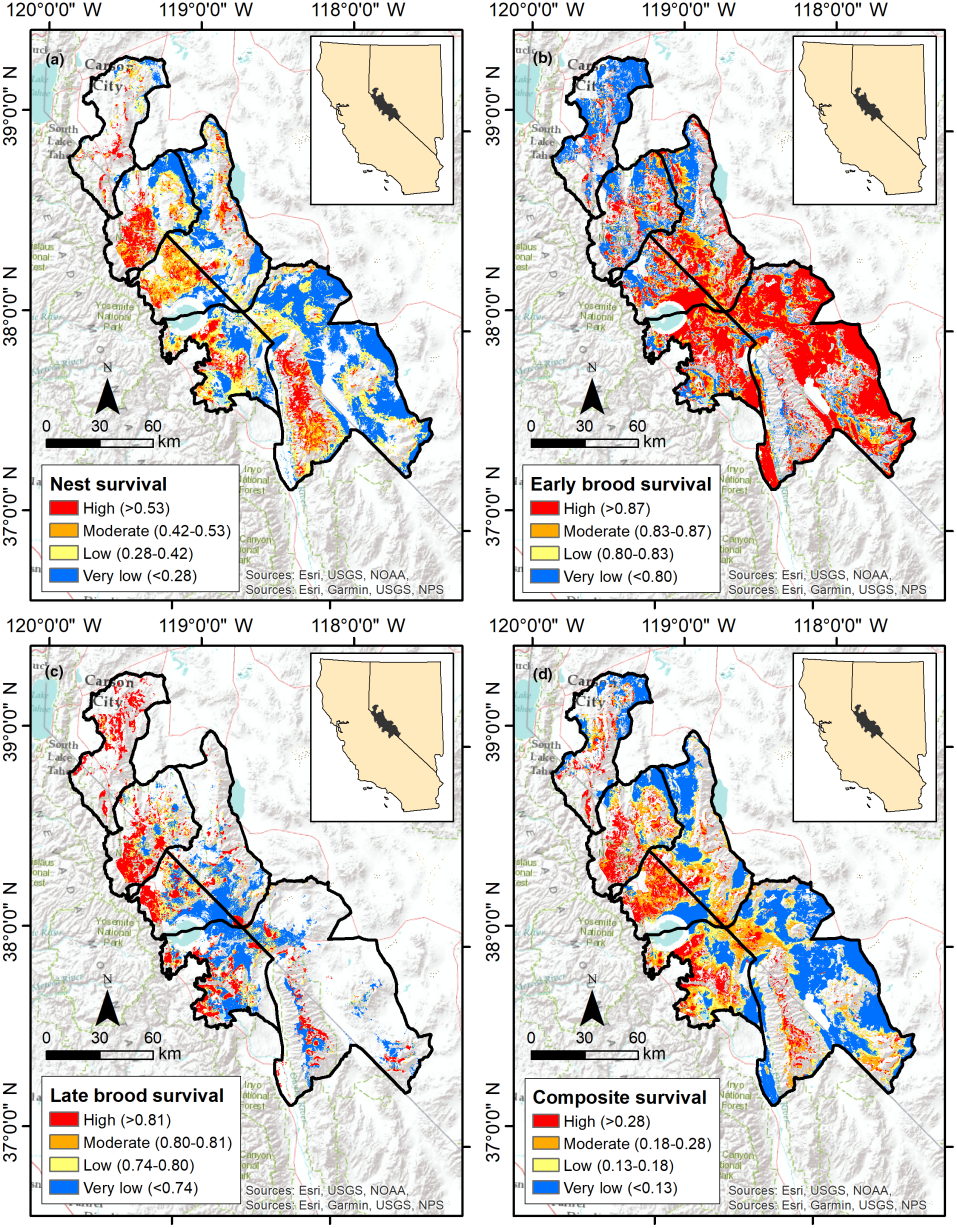
Maps of categorized survival for greater sage‐grouse in the Bi‐State Distinct Population Segment from 2003 to 2019 during nesting (a), early brood‐rearing (b), late brood‐rearing (c), and a composite survival index of the 3 reproductive life stages (d). Survival rates are noted parenthetically. Survival was not evaluated in areas considered nonhabitat based on selection.

**FIGURE 5:**
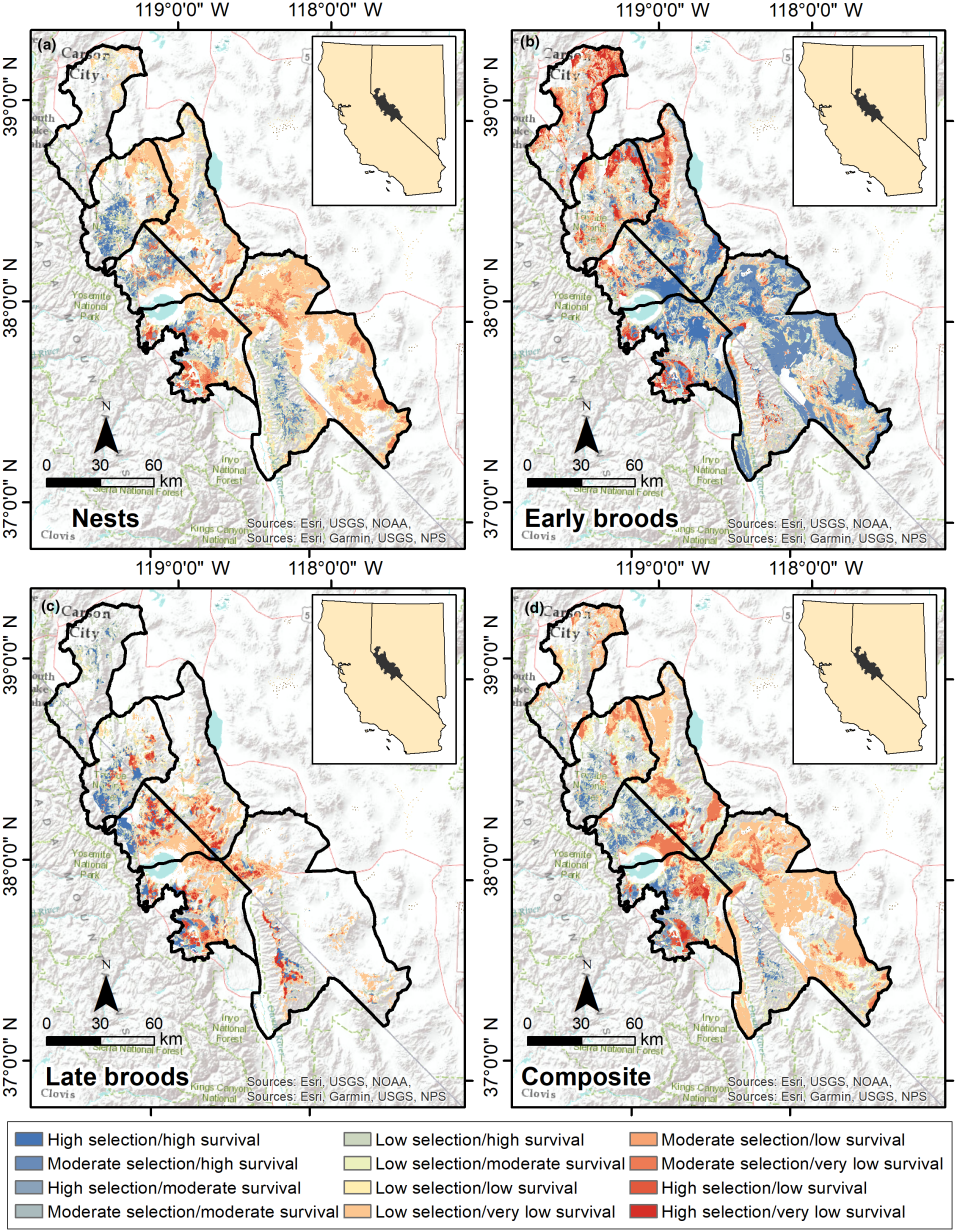
Source‐sink maps for greater sage‐grouse in the Bi‐State Distinct Population Segment from 2003 to 2019 during nesting (a), early brood rearing (b), late brood rearing (c) and a composite of the 3 reproductive life stages (d). Habitat is ranked from high (blue; high or moderate selection and high or moderate survival) to low (red; high or moderate selection and low or very low survival), representing source and sink habitats, respectively.

We mapped selection (Figure 3) and survival (Figure 4) across the Bi‐State DPS. We calculated composite selection and survival indices across the Bi‐State DPS by combining the selection and survival maps for the 3 reproductive life stages. Based on the composite selection index, a total of 5.3% of the Bi‐State region was classified as high selection (844 km^2^), 15.2% as moderate (2419 km^2^), 41.2% as low (6536 km^2^), and 38.3% as nonhabitat (6325 km^2^; Figure 3). Of the area classified as habitat, 17.2% of the Bi‐State region was classified as high survival (1684 km^2^), 19.9% as moderate (1947 km^2^), 14.8% as low (1445 km^2^), and 48.1% as very low (4621 km^2^; Figure 4). We also created combined selection‐survival maps across the Bi‐State DPS by combining categorized selection and survival maps. Productive habitat (high or moderate selection paired with high or moderate survival) represented 14.1% (1375 km^2^), whereas areas with potentially maladaptive selection (high or moderate selection paired with low or extremely low survival) represented 19.3% of the Bi‐State DPS (1888 km^2^; Figure 5). We also mapped specific example management categories using the composite selection map and the abundance and space use index (see Appendix S2).

## 4 DISCUSSION

Resource selection analyses and associated maps are often used by managers to guide conservation actions (Crawford et al., 2020; Pratt & Beck, 2021; Saher et al., 2022). However, the designation of important habitat solely based on species occupancy or use can be misleading. Incorporating demographic measures such as reproductive success provides increased power and detail for ranking habitat for management priority, particularly across multiple life stages and large spatial extents (Gibson et al., 2016; Pratt & Beck, 2021; Stephens et al., 2015). We provide a quantitative approach to differentiate productive habitats supporting high selection and survival from areas of maladaptive selection where selection and survival are misaligned at large spatial scales. These combined selection‐survival maps can provide a more complete understanding of habitat relationships and improve prioritization of important areas (Gibson et al., 2016; Holbrook et al., 2017; Pratt & Beck, 2021). By combining an understanding of potential habitat based on the composite selection index, which provides information on used areas, with knowledge of areas supporting improved demographic performance based on the composite survival index, our results illuminated productive habitat that supported both high selection and high survival, thus providing critical value for long‐term conservation (Crawford et al., 2020; O'Neil et al., 2020; Saher et al., 2022). In contrast, areas with maladaptive habitat selection, highlighted in our approach as areas with high selection but corresponding low reproductive success, could represent sink habitat for a given life stage (e.g., nesting sinks), possibly contributing to declines in overall population performance (O'Neil et al., 2020). Identified potential sink habitats represent important areas to evaluate specific mechanisms leading to poor reproductive performance and the potential for habitat improvement or restoration to improve the demographic rates (Holbrook et al., 2017). In particular, the variation in the amount of sink habitat across life stages could have implications for where habitat improvement would be best targeted. Simply evaluating selection could lead to management decisions that inadvertently promote relatively poor‐quality habitat that supports lower survival. In contrast, focusing solely on survival may not increase local distribution, as individuals need to select these areas first to have a population‐level impact. Our approach was then combined with information on species occupancy from the abundance and space use index (see Appendix S2; Coates, Casazza, et al., 2016), to further inform the targeting of management actions, such as reintroduction, in areas that have high potential habitat quality but minimal occupancy. Our combined approach, evaluated across multiple reproductive life stages at a landscape scale, can easily be adapted to other species. We provide two examples from our sage‐grouse case study to illustrate the importance of evaluating both selection and survival to highlight mismatches and trade‐offs.

### 4.1 Wildfire and annual grass

Wildfire is currently reshaping ecosystems across western North America (Flannigan et al., 2009), and our results suggest mismatches in selection and survival consequences for burned areas. During brood rearing, sage‐grouse avoided burned areas despite experiencing higher survival in those areas. However, only 2% of nests were within fire perimeters, and the apparent avoidance during the brood‐rearing period may be partially due to a lack of fires in areas accessible to sage‐grouse broods. While previous studies have found negative effects of wildfire on sage‐grouse across multiple life stages (Anthony et al., 2022; O'Neil et al., 2020), this has largely been attributed to recent megafires (Anthony et al., 2022; Dudley et al., 2021) coupled with the feedback loop between fire and invasive annual grasses, which has had the strongest negative effects in other parts of the Great Basin (Brussee et al., 2022; Coates, Ricca, et al., 2016; Germino et al., 2016). In comparison, megafires are rare in the Bi‐State, and invasion by annual grasses is uncommon (only 4.5% of burned areas had >10% annual grass cover). In addition, previous studies have found positive short‐term effects of wildfire on brood survival when cheatgrass is not present, likely due to increased herbaceous cover and forb production (Brussee et al., 2022), which is consistent with our findings. Given the large‐scale threat posed by wildfire in sagebrush ecosystems, identifying productive habitats that support high selection and high survival across multiple life stages, especially with respect to burned areas, will likely be key to guiding habitat management under changing future conditions.

### 4.2 Conifer cover

Increased conifer cover has negative consequences for the population performance of sage‐grouse (Olsen et al., 2021), and our results indicated strong avoidance of even low amounts of pinyon‐juniper cover across reproductive life stages. During nesting, sage‐grouse strongly avoided low levels of pinyon‐juniper encroachment, but the strength of the avoidance decreased during early and late brood‐rearing. Adult survival was lower in areas with more conifer cover (Coates et al., 2017), while conifer removal improved brood success (Sandford et al., 2017). Particularly at low levels of encroachment when shrubs are still dominant, conifer cover can represent an ecological trap where resources appear attractive but survival is reduced, in part due to the increased presence of predators such as perching raptors (Coates et al., 2017). Due to strong avoidance across all life stages, however, we found little to no evidence of an effect of conifer cover on survival, although there was high uncertainty around the effect on early brood survival. Reduced avoidance later in the season is likely due to trade‐offs where broods must move up in elevation to access limited mesic areas that provide important cover and forage resources (Saher et al., 2022). However, high‐elevation areas also tend to have greater pinyon‐juniper cover, thus making it more difficult for broods to avoid conifers, potentially with negative survival consequences, although our results did not support impacts on survival. Overall, this suggests that sage‐grouse may face important trade‐offs during reproductive life stages, wherein avoidance of risk from predators may be prioritized during nesting and early brood rearing. Access to forage resources, such as those in high‐elevation mesic areas, is likely most important during the late brood‐rearing period when chicks are larger, risk is less of a factor, and resources become increasingly limited. If such trade‐offs are highlighted and accounted for, our results suggest targeted restoration activities such as conifer removal in places like high‐elevation mesic areas that birds are selecting for but where they are experiencing reduced survival would be highly beneficial for sage‐grouse.

Our study was not without limitations. Perhaps most importantly, we did not incorporate movement dynamics because we only looked at how individual pixels were ranked across different life stages. Nevertheless, individuals that do not have to move large distances to meet their life stage habitat requirements are likely to be more successful, and our analysis identifies areas that benefit multiple reproductive life stages, allowing us to highlight both mismatches between selection and survival and trade‐offs between life stages. In addition, we modeled brood survival, which is a binary presence/absence data type, rather than chick survival, which inevitably leads to a loss of information that could confound our results. When possible, modeling chick survival would be preferred, although marking and tracking individual chicks comes with additional challenges that we could not overcome in this study. We also lacked sufficient data on some features to reliably estimate effects, in large part due to a lack of those features on the landscape within our study area, but that inevitably limited our inferences regarding specific features. For example, only 2% of nests were within fire perimeters, and no nests or broods were located near agricultural cropland. In addition, some temporally varying covariates, such as precipitation, invariably affect habitat quality but are difficult to adequately capture in a habitat selection analysis. However, evaluating both habitat selection and survival across many years, as in our study, should provide useful relative indices across time. Finally, by limiting our study to the breeding season, we were only able to identify areas that may represent sinks for a single life stage rather than population‐level sinks. Nonetheless, reproduction has a large impact on sage‐grouse population dynamics (Taylor et al., 2012), and we would expect areas supporting lower survival during the nesting and brood‐rearing periods to have correspondingly lower population growth rates.

## 5 CONCLUSIONS

By evaluating both habitat selection and survival across multiple key reproductive life stages for sage‐grouse, we were able to highlight a more complete picture of life history requirements and constraints for this species of high conservation concern, and our approach can easily be applied to any species for which habitat selection and survival information are available to guide management actions. Using widely available remotely sensed data, managers can then evaluate selection and survival across large spatial extents and target appropriate approaches for conservation and restoration in imperiled ecosystems. For example, productive areas that support both high habitat selection and high survival may be more amenable for conservation and protection efforts. In contrast, areas of maladaptive habitat selection (i.e., high selection but low survival) may be better targeted for restoration efforts to improve habitat and subsequent demographic performance. In addition, maps of habitat selection and survival can be combined with measures of current occupancy to guide management actions such as reintroductions, which might be more successful in priority areas that have high habitat quality based on both habitat selection and survival but are currently unoccupied. Overall, we demonstrate a straightforward quantitative approach for ranking habitat that incorporates metrics of both selection and demographic performance that can be used to tailor management to specific areas.

## AUTHOR CONTRIBUTIONS

**Megan C. Milligan:** Conceptualization (equal); data curation (equal); formal analysis (lead); methodology (equal); writing – original draft (lead). **Peter S. Coates:** Conceptualization (lead); methodology (equal); resources (lead); supervision (lead); writing – review and editing (equal). **Brianne E. Brussee:** Conceptualization (equal); data curation (equal); methodology (equal); writing – review and editing (equal). **Shawn T. O'Neil:** Conceptualization (equal); methodology (equal); supervision (equal); writing – review and editing (equal). **Steven R. Mathews:** Conceptualization (equal); writing – review and editing (equal). **Shawn P. Espinosa:** Conceptualization (equal); writing – review and editing (equal). **Katherine Miller:**Conceptualization (equal); writing – review and editing (equal). **Daniel Skalos:** Conceptualization (equal); writing – review and editing (equal). **Lief A. Wiechman:** Conceptualization (equal); writing – review and editing (equal). **Steve Abele:** Conceptualization (equal); writing – review and editing (equal). **John Boone:** Conceptualization (equal); writing – review and editing (equal). **Kristie Boatner:** Conceptualization (equal); writing – review and editing (equal). **Heather Stone:** Conceptualization (equal); writing – review and editing (equal). **Michael L. Casazza:** Conceptualization (equal); writing – review and editing (equal).

## Supporting information


Appendix S1 and S2.

## Data Availability

Data supporting the results of this manuscript are available at Coates et al. (2024).

## References

[ece310891-bib-0001] Aldridge, C. L. , & Boyce, M. S. (2007). Linking occurrence and fitness to persistence: Habitat‐based approach for endangered greater sage‐grouse. Ecological Applications, 17(2), 508–526. 17489256 10.1890/05-1871

[ece310891-bib-0002] Ammann, G. A. (1944). Determining the age of pinnated and sharp‐tailed grouses. Journal of Wildlife Management, 8(2), 170–171.

[ece310891-bib-0003] Anthony, C. R. , Foster, L. J. , Hagen, C. A. , & Dugger, K. M. (2022). Acute and lagged fitness consequences for a sagebrush obligate in a post mega‐wildfire landscape. Ecology and Evolution, 12(1), e8488. 35127022 10.1002/ece3.8488 PMC8794719

[ece310891-bib-0005] Avgar, T. , Lele, S. R. , Keim, J. L. , & Boyce, M. S. (2017). Relative selection strength: Quantifying effect size in habitat‐ and step‐selection inference. Ecology and Evolution, 7(14), 5322–5330. 10.1002/ece3.3122 28770070 PMC5528224

[ece310891-bib-0006] Battin, J. (2004). When good animals love bad habitats: Ecological traps and the conservation of animal populations. Conservation Biology, 18(6), 1482–1491. 10.1111/j.1523-1739.2004.00417.x

[ece310891-bib-0007] Blomberg, E. J. , Sedinger, J. S. , Gibson, D. , Coates, P. S. , & Casazza, M. L. (2014). Carryover effects and climatic conditions influence the postfledging survival of greater sage‐grouse. Ecology and Evolution, 4(23), 4488–4499. 25512845 10.1002/ece3.1139 PMC4264898

[ece310891-bib-0008] Boyce, M. S. (2006). Scale for resource selection functions. Diversity and Distributions, 12(3), 269–276. 10.1111/j.1366-9516.2006.00243.x

[ece310891-bib-0009] Boyte, S. P. , & Wylie, B. K. (2016). Near‐real‐time cheatgrass percent cover in the northern Great Basin, USA, 2015. Rangelands, 38(5), 278–284.

[ece310891-bib-0010] Brussee, B. E. , Coates, P. S. , O'Neil, S. T. , Espinosa, S. P. , Gardner, S. C. , Casazza, M. L. , & Delehanty, D. J. (2022). Wildfire‐cheatgrass cycles are linked to maladaptive habitat selection in a sagebrush indicator species. Global Ecology and Conservation, 167(231–244), e02147.

[ece310891-bib-0011] Caro, T. , Rowe, Z. , Berger, J. , Wholey, P. , & Dobson, A. (2022). An inconvenient misconception: Climate change is not the principal driver of biodiversity loss. Conservation Letters, 15(3), e12868.

[ece310891-bib-0012] Coates, P. S. , Brussee, B. E. , Ricca, M. A. , Severson, J. P. , Casazza, M. L. , Gustafson, K. B. , Espinosa, S. P. , Gardner, S. C. , & Delehanty, D. J. (2020). Spatially explicit models of seasonal habitat for greater sage‐grouse at broad spatial scales: Informing areas for management in Nevada and northeastern California. Ecology and Evolution, 10, 104–118. 10.1002/ece3.5842 31993115 PMC6972839

[ece310891-bib-0013] Coates, P. S. , Casazza, M. L. , Ricca, M. A. , Brussee, B. E. , Blomberg, E. J. , Gustafson, K. B. , Overton, C. T. , Davis, D. M. , Niell, L. E. , Espinosa, S. P. , Gardner, S. C. , & Delehanty, D. J. (2016). Integrating spatially explicit indices of abundance and habitat quality: An applied example for greater sage‐grouse management. Journal of Applied Ecology, 53, 83–95. 10.1111/1365-2664.12558 26877545 PMC4737303

[ece310891-bib-0045] Coates, P. S. , Milligan, M. C. , Brussee, B. E. , O'Neil, S. T. , & Chenaille, M. P. (2024). Rasters and tables for selection and survival of Greater Sage‐grouse nests and broods in the Bi‐State Distinct Population Segment of California and Nevada. *U.S. Geological Survey Data Release*. 10.5066/P95HTJG8

[ece310891-bib-0014] Coates, P. S. , Prochazka, B. G. , Ricca, M. A. , Gustafson, K. B. , Ziegler, P. , & Casazza, M. L. (2017). Pinyon and juniper encroachment into sagebrush ecosystems impacts distribution and survival of greater sage‐grouse. Rangeland Ecology & Management, 70, 25–38. 10.1016/j.rama.2016.09.001

[ece310891-bib-0015] Coates, P. S. , Ricca, M. A. , Prochazka, B. G. , Brooks, M. L. , Doherty, K. E. , Kroger, T. , Blomberg, E. J. , Hagen, C. A. , Casazza, M. L. , Coates, P. S. , Ricca, M. A. , Prochazka, B. G. , Brooks, M. L. , Doherty, K. E. , & Kroger, T. (2016). Wildfire, climate, and invasive grass interactions negatively impact an indicator species by reshaping sagebrush ecosystems. Proceedings of the National Academy of Sciences of the United States of America, 113(48), 12745–12750. 10.1073/pnas.1617905113 27791084 PMC5111658

[ece310891-bib-0016] Coates, P. S. , Ricca, M. A. , Prochazka, B. G. , O'Neil, S. T. , Severson, J. P. , Mathews, S. R. , Espinosa, S. P. , Gardner, S. C. , Lisius, S. , & Delehanty, D. J. (2020). Population and habitat analyses for greater sage‐grouse (*Centrocercus urophasianus*) in the Bi‐State Distinct Population Segment – 2018 update. *U*.*S*. *Geological Survey Open‐File Report 2019‐1149*.

[ece310891-bib-0017] Crawford, B. A. , Maerz, J. C. , & Moore, C. T. (2020). Expert‐informed habitat suitability analysis for at‐risk species assessment and conservation planning. Journal of Fish and Wildlife Management, 11(1), 130–150. 10.3996/092019-JFWM-075

[ece310891-bib-0018] Doherty, K. E. , Evans, J. S. , Coates, P. S. , Juliusson, L. M. , & Fedy, B. C. (2016). Importance of regional variation in conservation planning: A rangewide example of the greater sage‐grouse. Ecosphere, 7(10), e01462. 10.1002/ecs2.1462

[ece310891-bib-0019] Dudko, J. E. , Coates, P. S. , & Delehanty, D. J. (2018). Movements of female sage grouse *Centrocercus urophasianus* during incubation recess. IBIS, 161(1), 222–229. 10.1111/ibi.12670

[ece310891-bib-0020] Dudley, I. F. , Coates, P. S. , Prochazka, B. G. , O'Neil, S. T. , Gardner, S. , & Delehanty, D. J. (2021). Large‐scale wildfire reduces population growth in a peripheral population of sage‐grouse. Fire Ecology, 17(1), 1–13.

[ece310891-bib-0021] Evans, J. S. , & Oakleaf, J. (2012). *Geomorphometry & gradient metrics toolbox* (*ArcGIS 10*.*0*).

[ece310891-bib-0022] Fieberg, J. R. , Forester, J. D. , Street, G. M. , Johnson, D. H. , ArchMiller, A. A. , & Matthiopoulos, J. (2018). Used‐habitat calibration plots: A new procedure for validating species distribution, resource selection, and step‐selection models. Ecography, 41(5), 737–752. 10.1111/ecog.03123

[ece310891-bib-0023] Flannigan, M. D. , Krawchuk, M. A. , de Groot, W. J. , Wotton, B. M. , & Gowman, L. M. (2009). Implications of changing climate for global wildland fire. International Journal of Wildland Fire, 18(5), 483–507.

[ece310891-bib-0024] Fretwell, S. D. , & Lucas, H. L. (1970). On territorial behavior and other factors influencing habitat distribution in birds. I. Theoretical development. Acta Biotheoretica, 19, 16–36.

[ece310891-bib-0025] Gelman, A. , & Hill, J. (2006). Data analysis using regression and multilevel/hierarchical models. Cambridge University Press.

[ece310891-bib-0026] Gelman, A. , Stern, H. S. , Carlin, J. B. , Dunson, D. B. , Vehtari, A. , & Rubin, D. B. (2013). Bayesian data analysis (3rd ed.). CRC Press.

[ece310891-bib-0027] Germino, M. J. , Belnap, J. , Stark, J. M. , Allen, E. B. , & Rau, B. M. (2016). Ecosystem impacts of exotic annual invaders in the genus Bromus. In J. C. Chambers (Ed.), Exotic brome‐grasses in arid and semiarid ecosystems of the Western US (pp. 61–95). Springer. 10.1007/978-3-319-24930-8

[ece310891-bib-0028] Gibson, D. , Blomberg, E. J. , Atamian, M. T. , & Sedinger, J. S. (2016). Nesting habitat selection influences nest and early offspring survival in greater sage‐grouse. The Condor: Ornithological Applications, 118(4), 689–702. 10.1650/CONDOR-16-62.1

[ece310891-bib-0029] Gustafson, K. B. , Coates, P. S. , Roth, C. L. , Chenaille, M. P. , Ricca, M. A. , Sanchez‐Chopitea, E. , & Casazza, M. L. (2018). Using object‐based image analysis to conduct high‐resolution conifer extraction at regional spatial scales. International Journal of Applied Earth Observation and Geoinformation, 73, 148–155.

[ece310891-bib-0030] Hanser, S. E. , & Knick, S. T. (2011). Greater sage‐grouse as an umbrella species for shrubland passerine birds: A multiscale assessment. In S. T. Knick & J. W. Connelly (Eds.), Greater sage‐grouse: Ecology and conservation of a landscape species and its habitats (pp. 474–487). University of California Press. 10.1525/california/9780520267114.003.0020

[ece310891-bib-0031] Hanski, I. , & Gilpin, M. (1991). Metapopulation dynamics. Biological Journal of the Linnean Society, 42, 3–16. 10.1111/j.1095-8312.1991.tb00548.x

[ece310891-bib-0032] Heinrichs, J. A. , Bender, D. J. , Gummer, D. L. , & Schumaker, N. H. (2010). Assessing critical habitat: Evaluating the relative contribution of habitats to population persistence. Biological Conservation, 143, 2229–2237. 10.1016/j.biocon.2010.06.009

[ece310891-bib-0033] Holbrook, J. D. , Squires, J. R. , Olson, L. E. , Decesare, N. J. , & Lawrence, R. L. (2017). Understanding and predicting habitat for wildlife conservation: The case of Canada lynx at the range periphery. Ecosphere, 8(9), e01939. 10.1002/ecs2.1939

[ece310891-bib-0034] Hooten, M. B. , & Hobbs, N. T. (2015). A guide to Bayesian model selection for ecologists. Ecological Monographs, 85(1), 3–28.

[ece310891-bib-0035] Howe, R. W. , Davis, G. J. , & Mosca, V. (1991). The demographic significance of ‘sink’ populations. Biological Conservation, 57(3), 239–255.

[ece310891-bib-0036] Johnson, C. J. , Nielsen, S. E. , Merrill, E. H. , McDonald, T. L. , & Boyce, M. S. (2006). Resource selection functions based on use‐availability data: Theoretical motivation and evaluation methods. Journal of Wildlife Management, 41, 347–357.

[ece310891-bib-0037] Johnson, M. D. (2007). Measuring habitat quality: A review. The Condor: Ornithological Applications, 109, 489–504. 10.1650/8347.1

[ece310891-bib-0038] Jones, J. (2001). Habitat selection studies in avian ecology: A critical review. The Auk: Ornithological Advances, 118(2), 557–562.

[ece310891-bib-0039] Kéry, M. (2010). Introduction to WinBUGS for ecologists: Bayesian approach to regression, ANOVA, mixed models and related analyses. Academic Press.

[ece310891-bib-0040] Kristan, W. B., III . (2003). The role of habitat selection behavior in population dynamics: Source–sink systems and ecological traps. Oikos, 103(3), 457–468.

[ece310891-bib-0041] Makowski, D. , Ben‐Shachar, M. S. , Chen, S. H. , & Lüdecke, D. (2019). Indices of effect existence and significance in the Bayesian framework. Frontiers in Psychology, 10, 2767. 10.3389/fpsyg.2019.02767 31920819 PMC6914840

[ece310891-bib-0042] Matthiopoulos, J. , Fieberg, J. , Aarts, G. , Beyer, H. L. , Morales, J. M. , & Haydon, D. T. (2015). Establishing the link between habitat selection and animal population dynamics. Ecological Monographs, 85(3), 413–436. 10.1890/14-2244.1

[ece310891-bib-0043] McDonald, T. L. (2013). The point process use‐availability or presence‐only likelihood and comments on analysis. Journal of Animal Ecology, 82(6), 1174–1182. 10.1111/1365-2656.12132 24111555

[ece310891-bib-0044] Merkle, J. A. , Abrahms, B. , Armstrong, J. B. , Sawyer, H. , Costa, D. P. , & Chalfoun, A. D. (2022). Site fidelity as a maladaptive behavior in the Anthropocene. Frontiers in Ecology and the Environment, 20(3), 187–194.

[ece310891-bib-0004] Millennium Ecosystem Assessment . (2005). Ecosystems and human well‐being synthesis. Island Press.

[ece310891-bib-0046] Northrup, J. M. , Hooten, M. B. , Anderson, C. R. , & Wittemyer, G. (2013). Practical guidance on characterizing availability in resource selection functions under a use‐availability design. Ecology, 94(7), 1456–1463. 10.1890/12-1688.1 23951705

[ece310891-bib-0047] Noss, R. F. , La Roe, E. T., III , & Scott, J. M. (1995). Endangered ecosystems of the United States: A preliminary assessment of loss and degradation. *Biological Report 28*, *U*.*S*. *Department of the Interior, National Biological Service* .

[ece310891-bib-0048] O'Neil, S. T. , Coates, P. S. , Brussee, B. E. , Ricca, M. A. , Espinosa, S. P. , Gardner, S. C. , & Delehanty, D. J. (2020). Wildfire and the ecological niche: Diminishing habitat suitability for an indicator species within semi‐arid ecosystems. Global Change Biology, 26, 6296–6312. 10.1111/gcb.15300 32741106 PMC7693117

[ece310891-bib-0049] Olsen, A. C. , Severson, J. P. , Maestas, J. D. , Naugle, D. E. , Smith, J. T. , Tack, J. D. , Yates, K. H. , & Hagen, C. A. (2021). Reversing tree expansion in sagebrush steppe yields population‐level benefit for imperiled grouse. Ecosphere, 12(6), e03551.

[ece310891-bib-0050] Park, T. , & Casella, G. (2008). The Bayesian lasso. Journal of the American Statistical Association, 103(482), 681–686. 10.1198/016214508000000337

[ece310891-bib-0051] Plummer, M. (2003). JAGS: A program for analysis of Bayesian graphical models using Gibbs sampling. In *Proceedings of the 3rd International Workshop on Distributed Statistical Computing* (p. 125).

[ece310891-bib-0052] Pratt, A. C. , & Beck, J. L. (2021). Do greater sage‐grouse exhibit maladaptive habitat selection? Ecosphere, 12(3), e03354. 10.1002/ecs2.3354

[ece310891-bib-0053] Pulliam, H. R. (1988). Sources, sinks, and population regulation. The American Naturalist, 132(5), 652–661.

[ece310891-bib-0074] R Core Team . (2021). R–The R project for statistical computing. R Foundation for Statistical Computing. https://www.r‐project.org/

[ece310891-bib-0054] Rigge, M. , Bunde, B. , Shi, H. , & Postma, K. (2021). Rangeland Condition Monitoring Assessment and Projection (RCMAP) fractional component time‐series across the Western U.S. 1985–2020. *US Geological Survey Data Release*, 10.5066/p95iq4bt

[ece310891-bib-0055] Rigge, M. , Homer, C. , Cleeves, L. , Meyer, D. , Bunde, B. , Shi, H. , Xian, G. , & Bobo, M. (2020). Quantifying western US rangelands as fractional components with multi‐resolution remote sensing and in situ data. Remote Sensing, 12, 412.

[ece310891-bib-0056] Robertson, B. A. , & Hutto, R. L. (2006). A framework for understanding ecological traps and an evaluation of existing evidence. Ecology, 87(5), 1075–1085. 16761584 10.1890/0012-9658(2006)87[1075:affuet]2.0.co;2

[ece310891-bib-0057] Saher, D. J. , O'Donnell, M. S. , Aldridge, C. L. , & Heinrichs, J. A. (2022). Balancing model generality and specificity in management‐focused habitat selection models for Gunnison sage‐grouse. Global Ecology and Conservation, 35, e01935. 10.1016/j.gecco.2021.e01935

[ece310891-bib-0058] Sandford, C. P. , Kohl, M. T. , Messmer, T. A. , Dahlgren, D. K. , Cook, A. , & Wing, B. R. (2017). Greater sage‐grouse resource selection drives reproductive fitness under a conifer removal strategy. Rangeland Ecology & Management, 70(1), 59–67. 10.1016/j.rama.2016.09.002

[ece310891-bib-0059] Schmidt, J. H. , Walker, J. A. , Lindberg, M. S. , Johnson, D. S. , & Stephens, S. E. (2010). A general Bayesian hierarchical model for estimating survival of nests and young. The Auk, 127(2), 379–386.

[ece310891-bib-0060] Shaffer, T. L. (2004). A unified approach to analyzing nest success. The Auk, 121, 526–540. 10.1642/0004-8038(2004)121[0526:auatan]2.0.co;2

[ece310891-bib-0061] Sinnott, E. A. , Thompson, F. R., III , Weegman, M. D. , & Thompson, T. R. (2022). Northern bobwhite juvenile survival is greater in native grasslands managed with fire and grazing and lower in non‐native field borders and strip crop fields. The Condor, 124(1), duab057.

[ece310891-bib-0062] Stamps, J. A. , Krishnan, V. V. , & Reid, M. L. (2005). Search costs and habitat selection by dispersers. Ecology, 86(2), 510–518. 10.1890/04-0516

[ece310891-bib-0063] Stephens, P. A. , Pettorelli, N. , Barlow, J. , Whittingham, M. J. , & Cadotte, M. W. (2015). Management by proxy? The use of indices in applied ecology. Journal of Applied Ecology, 52(1), 1–6.

[ece310891-bib-0064] Stuber, E. F. , & Fontaine, J. J. (2019). How characteristic is the species characteristic selection scale? Global Ecology and Biogeography, 28, 1839–1854. 10.1111/geb.12998

[ece310891-bib-0065] Stuber, E. F. , Gruber, L. F. , & Fontaine, J. J. (2017). A Bayesian method for assessing multi‐scale species‐habitat relationships. Landscape Ecology, 32, 2365–2381. 10.1007/s10980-017-0575-y

[ece310891-bib-0066] Su, Y. S. , & Yajima, M. (2015). R2jags: A package for running jags from R. *R Package Version 0*.*05‐01*.

[ece310891-bib-0067] Taylor, R. L. , Walker, B. L. , Naugle, D. E. , & Mills, L. S. (2012). Managing multiple vital rates to maximize greater sage‐grouse population growth. Journal of Wildlife Management, 76(2), 336–347. 10.1002/jwmg.267

[ece310891-bib-0068] U.S. Fish and Wildlife Service . (2015). Endangered and threatened wildlife and plants; designation of critical habitat for the Bi‐state distinct population segment of greater sage‐grouse. Federal Register, 80, 59858–59942.

[ece310891-bib-0073] U.S. Fish and Wildlife Service . (2022). National Wetland Inventory.

[ece310891-bib-0069] U.S. Geological Survey . (2017a). National Hydrography Dataset. https://nhd.usgs.gov

[ece310891-bib-0070] U.S. Geological Survey . (2017b). The National Map: 3D Elevation Program.

[ece310891-bib-0071] Van Horne, B. (1983). Density as a misleading indicator of habitat quality. Journal of Wildlife Management, 47(4), 893–901.

