# Supplementary material for: Linking resource selection to population performance spatially to identify species' habitat across broad scales: An example of greater sage‐grouse in a distinct population segment
Source: Ecol Evol. 2024 Oct 10;14(10):e10891. doi: 10.1002/ece3.10891 (PMC11464893; doi:10.1002/ece3.10891)
Supplement: Supplementary file 1 — Appendix S1 and S2. [file ECE3-14-e10891-s001.docx]

**Linking resource selection to population performance spatially to identify species’ habitat across broad scales: an example of greater sage-grouse in a distinct population segment**

Megan C. Milligan^1^, Peter S. Coates^1^, Brianne E. Brussee^1^, Shawn T. O’Neil^1^, Steven R. Mathews^1^, Shawn P. Espinosa^2^, Daniel Skalos^3^, Lief A. Wiechman^4^, Steve Abele^5^, John Boone^6^, Kristie Boatner^7^, Heather Stone^8^, Michael L. Casazza^1^

^1^U.S. Geological Survey, Western Ecological Research Center, Dixon, CA

^2^Nevada Department of Wildlife, Reno, NV

^3^California Department of Fish and Wildlife, Sacramento, CA

^4^U.S. Geological Survey, Fort Collins Science Center, CO

^5^U.S. Fish and Wildlife Service, Reno, NV

^6^Great Basin Bird Observatory, Reno, NV

^7^U.S. Forest Service, Sparks, NV

^8^Bureau of Land Management, Bishop, CA

Any use of trade, firm, or product names is for descriptive purposes only and does not imply endorsement by the U.S. Government.

**APPENDIX S1**


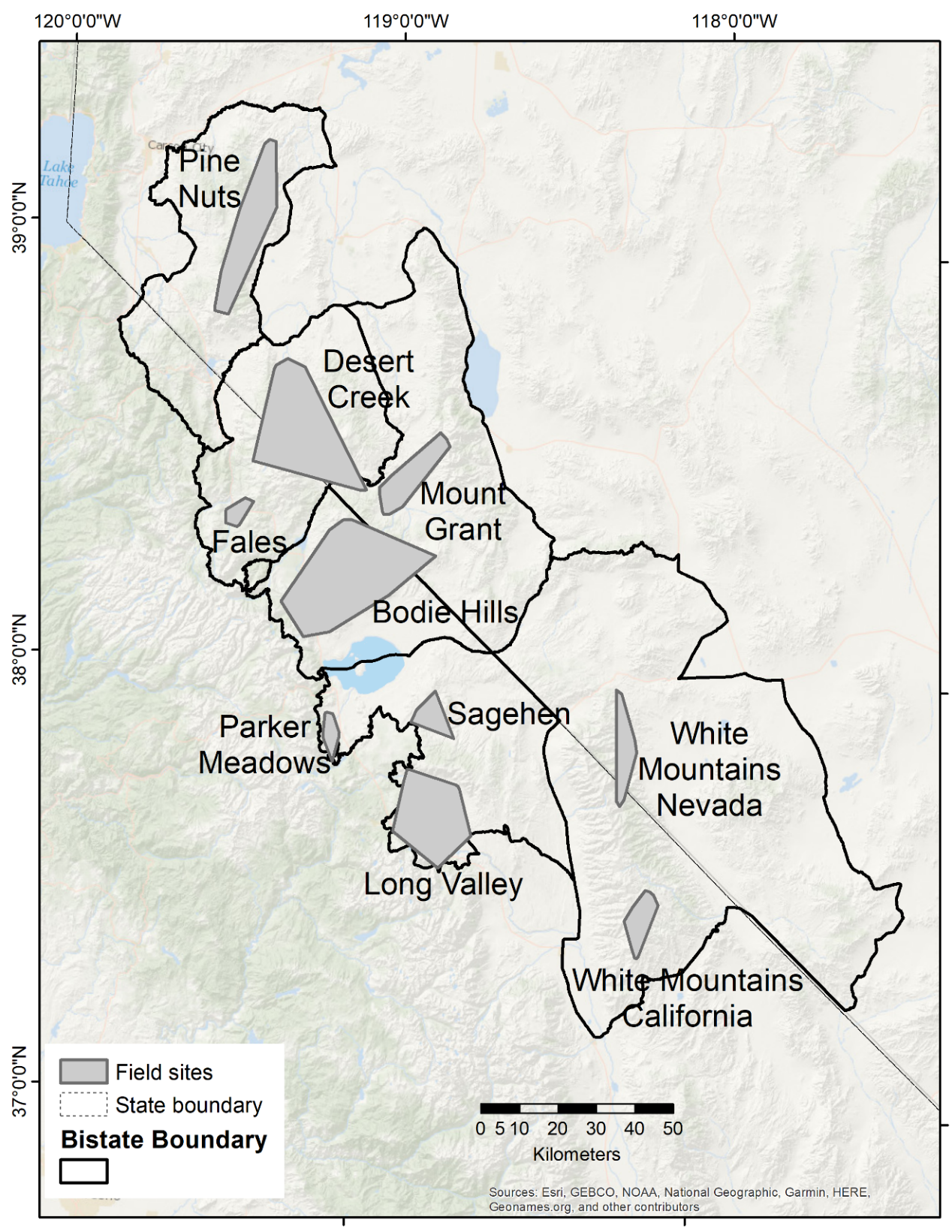


Figure S1. Sites in the Bi-State Distinct Population Segment where female greater sage-grouse were monitored from 2003–2019. Site boundaries represent minimum convex polygons calculated using nest and brood locations from the duration of the study.

**APPENDIX S2**


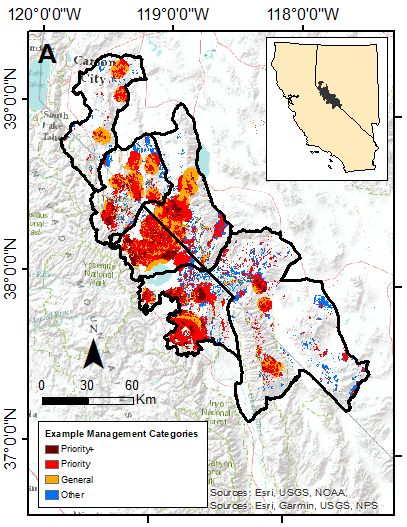


Figure S2. Example management categories based on selection of greater sage-grouse in the Bi-State Distinct Population Segment from 2003–2019.

| Table S1. Coefficient estimates for nest site selection and nest survival for greater sage-grouse in the Bi-State Distinct Population Segment from 2003 to 2019. The 95% credible intervals and the best selected scale for each group are noted parenthetically. | | | | | | |
| --- | --- | --- | --- | --- | --- | --- |
|  | **Nest selection** | | | **Nest survival** | | |
| **Group** | **Variable** | **β (95% CRI)** | **P(\|β\| > 0)** | **Variable** | **β (95% CRI)** | **P(\|β\| > 0)** |
| Shrubs | Shrub hgt. (75m) | 0.57 (0.39 ̶ 0.77) | 1.00 | Shrubs (75m) | 0.10 (-0.05 ̶ 0.27) | 0.89 |
| Herbaceous/ wet meadows | Herbaceous cover (167m) | -0.49 (-0.70 ̶ -0.29) | 1.00 | Dist. to wet meadow | 0.03 (-0.22 ̶ 0.38) | 0.63 |
| Annual grass | Annual grass (1451m) | -0.06 (-0.24 ̶ 0.10) | 0.76 | Annual grass (1451m) | 0.01 (-0.11 ̶ 0.15) | 0.56 |
| Pinyon-juniper | CC1 (260m) | -1.38 (-1.77 ̶ -1.02) | 1.00 | Dist. to CC2 | 0.06 (-0.19 ̶ 0.46) | 0.69 |
| Bare ground | Bare ground (167m) | -0.34 (-0.58 ̶ -0.10) | 1.00 | Bare ground (75m) | -0.02 (-0.20 ̶ 0.13) | 0.63 |
| Burned area | CBA (260m) | -0.11 (-0.28 ̶ 0.03) | 0.93 | CBA (1451m) | 0.13 (-0.01 ̶ 0.31) | 0.97 |
| Streams | Perennial streams (439m) | -0.46 (-0.66 ̶ -0.27) | 1.00 | Dist. to intermittent stream | 0.09 (-0.16 ̶ 0.55) | 0.76 |
| Springs | Springs (1451m) | -0.21 (-0.34 ̶ -0.08) | 1.00 | Springs (439m) | 0.05 (-0.05 ̶ 0.21) | 0.83 |
| Roughness | Slope (260m) | -0.04 (-0.23 ̶ 0.13) | 0.68 | Roughness (439m) | 0.10 (-0.04 ̶ 0.30) | 0.91 |
| Terrain | Transformed aspect (1451m) | -0.23 (-0.37 ̶ -0.10) | 1.00 | CTI (260m) | -0.07 (-0.25 ̶ 0.07) | 0.83 |
| Elevation | Elevation (167m) | 0.27 (0.05 ̶ 0.47) | 0.99 | Elevation (1451m) | 0.09 (-0.06 ̶ 0.28) | 0.86 |
| Saline lakes | Saline lakes (5,000m) | -0.57 (-2.35 ̶ 0.01) | 0.97 |  |  |  |

| Table S2. Coefficient estimates for early brood selection and survival for greater sage-grouse in the Bi-State Distinct Population Segment from 2003 to 2019. The 95% credible intervals and the best selected scale for each group are noted parenthetically. | | | | | | |
| --- | --- | --- | --- | --- | --- | --- |
|  | **Early brood selection** | | | **Early brood survival** | | |
| **Group** | **Variable** | **β (95% CRI)** | **P(\|β\| > 0)** | **Variable** | **β (95% CRI)** | **P(\|β\| > 0)** |
| Shrubs | Sage hgt. (75m) | 0.38 (0.10 ̶ 0.68) | 1.00 | Shrubs (370m) | -0.10 (-0.55 ̶ 0.18) | 0.91 |
| Herbaceous/ wet meadows | Wet meadows (75m) | -0.22 (-0.63 ̶ 0.05) | 0.93 | Wet meadows (260m) | -0.11 (-0.49 ̶ 0.16) | 0.75 |
| Annual grass | Annual grass (1451m) | 0.10 (-0.16 ̶ 0.36) | 0.78 | Annual grass (260m) | -0.22 (-0.57 ̶ 0.08) | 0.57 |
| Conifer cover | CC1 (167m) | -0.48 (-0.74 ̶ -0.24) | 1.00 | Dist. to CC2 | 0.11 (-0.30 ̶ 1.11) | 1.00 |
| Bare ground | Bare ground (260m) | 0.19 (-0.09 ̶ 0.51) | 0.91 | Bare ground (167m) | -0.06 (-0.50 ̶ 0.21) | 0.66 |
| Burned area | CBA (260m) | -0.17 (-0.41 ̶ 0.03) | 0.95 | CBA (439m) | 0.15 (-0.14 ̶ 0.98) | 0.86 |
| Streams | Perennial streams (1451m) | -0.36 (-0.65 ̶ -0.07) | 0.99 | Perennial streams (439m) | -0.05 (-0.40 ̶ 0.24) | 0.92 |
| Springs | Dist. to spring | -0.04 (-0.7 ̶ 0.56) | 0.57 | Springs (370m) | 0.33 (-0.07 ̶ 1.94) | 0.81 |
| Topography | Roughness (260m) | -0.98 (-1.54 ̶ -0.49) | 1.00 | Roughness (1451m) | 0.02 (-0.26 ̶ 0.37) | 0.79 |
| Temperature/ moisture | HLI (1451m) | -0.22 (-0.47 ̶ 0.01) | 0.97 | HLI (439m) | -0.37 (-0.64 ̶ -0.10) | 0.69 |
| Elevation | Elevation (75m) | 0.41 (0.14 ̶ 0.69) | 1.00 | Elevation (167m) | -0.16 (-0.54 ̶ 0.10) | 0.71 |
| Saline lakes | Saline lakes (5,000m) | -0.21 (-1.66 ̶ 0.12) | 0.82 |  |  |  |

| Table S3. Coefficient estimates for late brood selection and survival for greater sage-grouse in the Bi-State Distinct Population Segment from 2003 to 2019. The 95% credible intervals and the best selected scale for each group are noted parenthetically. | | | | | | |
| --- | --- | --- | --- | --- | --- | --- |
|  | **Late brood selection** | | | **Late brood survival** | | |
| **Group** | **Variable** | **β (95% CRI)** | **P(\|β\| > 0)** | **Variable** | **β (95% CRI)** | **P(\|β\| > 0)** |
| Shrubs | Sage hgt. (75m) | 0.50 (0.19 ̶ 0.82) | 1.00 | Shrub hgt. (1451m) | 0.08 (-0.24 ̶ 0.52) | 0.73 |
| Herbaceous/ wet meadows | Herbaceous cover (167m) | 0.35 (0.09 ̶ 0.62) | 1.00 | Herbaceous cover (1451m) | 0.08 (-0.22 ̶ 0.48) | 0.71 |
| Annual grass | Annual grass (260m) | 0.05 (-0.11 ̶ 0.22) | 0.74 | Annual grass (370m) | 0.05 (-0.18 ̶ 0.50) | 0.67 |
| Conifer cover | CC1 (370m) | -0.50 (-1.05 ̶ -0.03) | 0.98 | CC1 (370m) | -0.002 (-0.26 ̶ 0.28) | 0.49 |
| Bare ground | Bare ground (1451m) | -0.29 (-0.74 ̶ 0.09) | 0.93 | Bare ground (1451m) | -0.11 (-0.58 ̶ 0.22) | 0.76 |
| Burned area | CBA (1451m) | -0.25 (-0.52 ̶ 0.003) | 0.97 | CBA (370m) | 0.13 (-0.16 ̶ 0.85) | 0.78 |
| Streams | Perennial streams (1451m) | -0.29 (-0.56 ̶ -0.05) | 0.99 | Intermittent streams (1451m) | 0.33 (-0.05 ̶ 1.16) | 0.94 |
| Springs | Dist. to spring | -0.09 (-1.04 ̶ 0.71) | 0.61 | Springs (1451m) | 0.20 (-0.06 ̶ 0.67) | 0.92 |
| Topography | Slope (439m) | -1.94 (-2.55 ̶ -1.38) | 1.00 | Slope (1451m) | 0.24 (-0.09 ̶ 0.92) | 0.89 |
| Temperature/ moisture | Transformed aspect (75m) | -0.27 (-0.52 ̶ -0.03) | 0.99 | CTI (75m) | 0.18 (-0.10 ̶ 0.66) | 0.87 |
| Elevation | Elevation (370m) | 1.31 (0.82 ̶ 1.82) | 1.00 | Elevation (1451m) | -0.10 (-0.58 ̶ 0.16) | 0.77 |
| Saline lakes | Saline lakes (5,000m) | -0.41 (-2.43 ̶ 0.20) | 0.86 |  |  |  |

| Table S4. Candidate landscape covariates for habitat selection and survival models of greater sage-grouse in the Bi-State Distinct Population Segment from 2003 to 2019 for nest, early, and late brood-rearing periods, selected from a preliminary Bayesian latent indicator variable selection procedure, where covariates with greater proportion of the posterior distribution were selected over other grouped covariates. Numbers shown in bold text represent the covariates that were chosen for each group and analysis. | | | | | | | |
| --- | --- | --- | --- | --- | --- | --- | --- |
|  |  | Proportion of distribution | | | | | |
|  |  | Selection | | | Survival | | |
| Group | Covariates | Nest | Early brood | Late brood | Nest | Early brood | Late brood |
| Shrubs | Total shrub (%; 75 m) | 0 | 0.004 | 0.002 | **0.08** | 0.05 | 0.03 |
|  | Total shrub (%; 167 m) | 0 | 0.001 | 0.002 | 0.07 | 0.05 | 0.03 |
|  | Total shrub (%; 260 m) | 0 | 0.0018 | - | 0.07 | 0.05 | 0.03 |
|  | Total shrub (%; 370 m) | - | - | 0.002 | - | **0.054** | 0.03 |
|  | Total shrub (%; 439 m) | 0 | 0.001 | 0.004 | 0.05 | 0.05 | 0.03 |
|  | Total shrub (%; 1,451 m) | 0 | 0.001 | 0.002 | 0.04 | 0.04 | 0.04 |
|  | Total sagebrush (%; 75 m) | 0 | 0.03 | 0.07 | 0.05 | 0.03 | 0.03 |
|  | Total sagebrush (%; 167 m) | 0 | 0.002 | 0.07 | 0.05 | 0.03 | 0.04 |
|  | Total sagebrush (%; 260 m) | 0 | 0.001 | - | 0.04 | 0.04 | 0.03 |
|  | Total sagebrush (%; 370 m) | - | - | 0.08 | - | 0.03 | 0.03 |
|  | Total sagebrush (%; 439 m) | 0 | 0.001 | 0.1 | 0.05 | 0.04 | 0.03 |
|  | Total sagebrush (%; 1,451 m) | 0 | 0.001 | 0.14 | 0.05 | 0.05 | 0.03 |
|  | Shrub height (cm; 75 m) | **0.53** | 0.006 | 0.01 | 0.04 | 0.03 | 0.03 |
|  | Shrub height (cm; 167 m) | 0 | 0.002 | 0.008 | 0.04 | 0.04 | 0.04 |
|  | Shrub height (cm; 260 m) | 0 | 0.2 | - | 0.04 | 0.04 | 0.02 |
|  | Shrub height (cm; 370 m) | - | - | 0.007 | - | 0.05 | 0.03 |
|  | Shrub height (cm; 439 m) | 0 | 0.002 | 0.003 | 0.08 | 0.04 | 0.06 |
|  | Shrub height (cm; 1,451 m) | 0 | 0.001 | 0.002 | 0.06 | 0.04 | **0.09** |
|  | Sagebrush height (cm; 75 m) | 0.47 | **0.44** | **0.34** | 0.03 | 0.04 | 0.05 |
|  | Sagebrush height (cm; 167 m) | 0 | 0.12 | 0.12 | 0.04 | 0.04 | 0.05 |
|  | Sagebrush height (cm; 260 m) | 0 | 0.17 | - | 0.04 | 0.05 | 0.03 |
|  | Sagebrush height (cm; 370 m) | - | - | 0.008 | - | 0.04 | 0.03 |
|  | Sagebrush height (cm; 439 m) | 0 | 0.02 | 0.03 | 0.06 | 0.04 | 0.09 |
|  | Sagebrush height (cm; 1,451 m) | 0 | 0.002 | 0.01 | 0.05 | 0.04 | 0.07 |
| Herbaceous cover/wet meadows | Herbaceous cover (%; 75 m) | 0.001 | 0.07 | 0.2 | 0.09 | 0.03 | 0.06 |
|  | Herbaceous cover (%; 167 m) | **0.42** | 0.07 | **0.22** | 0.1 | 0.03 | 0.06 |
|  | Herbaceous cover (%; 260 m) | 0.04 | 0.08 | - | 0.09 | 0.03 | 0.7 |
|  | Herbaceous cover (%; 370 m) | - | - | 0.13 | - | 0.02 | 0.07 |
|  | Herbaceous cover (%; 439 m) | 0.002 | 0.07 | 0.12 | 0.09 | 0.02 | 0.07 |
|  | Herbaceous cover (%; 1,451 m) | 0 | 0.13 | 0.17 | 0.09 | 0.03 | **0.15** |
|  | Wet meadow density (wet meadow/km^2^; 75 m) | 0.005 | **0.16** | 0.004 | 0 | 0.08 | 0.06 |
|  | Wet meadow density (wet meadow/km^2^; 167 m) | 0.38 | 0.09 | 0.006 | 0.09 | 0.16 | 0.06 |
|  | Wet meadow density (wet meadow/km^2^; 260 m) | 0.14 | 0.09 | - | 0.11 | **0.2** | 0.07 |
|  | Wet meadow density (wet meadow/km^2^; 370 m) | - | - | 0.005 | - | 0.17 | 0.07 |
|  | Wet meadow density (wet meadow/km^2^; 439 m) | 0.008 | 0.08 | 0.007 | 0.1 | 0.18 | 0.08 |
|  | Wet meadow density (wet meadow/km^2^; 1,4541 m) | 0 | 0.08 | 0.01 | 0.08 | 0.03 | 0.07 |
|  | Distance to wet meadow (exponential | 0 | 0.11 | 0.14 | **0.15** | 0.03 | 0.1 |
| Bare ground | Bare ground cover (%; 75 m) | 0.15 | 0.12 | 0.21 | **0.38** | 0.17 | 0.09 |
|  | Bare ground cover (%; 167 m) | **0.52** | 0.16 | 0.15 | 0.19 | **0.19** | 0.11 |
|  | Bare ground cover (%; 260 m) | 0.26 | **0.26** | - | 0.17 | 0.17 | 0.13 |
|  | Bare ground cover (%; 370 m) | - | - | 0.15 | - | 0.16 | 0.11 |
|  | Bare ground cover (%; 439 m) | 0.05 | 0.24 | 0.19 | 0.14 | 0.16 | 0.12 |
|  | Bare ground cover (%; 1,451 m) | 0.02 | 0.22 | **0.3** | 0.14 | 0.16 | **0.45** |
| Annual grass | Annual grass cover(%; 260 m) | 0.33 | 0.35 | **0.33** | 0.32 | **0.32** | 0.21 |
|  | Annual grass cover(%; 370 m) | - | - | 0.28 | - | 0.24 | **0.37** |
|  | Annual grass cover(%; 439 m) | 0.33 | 0.29 | 0.2 | 0.32 | 0.22 | 0.25 |
|  | Annual grass cover(%; 1,451 m) | **0.34** | **0.36** | 0.2 | **0.37** | 0.25 | 0.17 |
| Conifer cover | Pinyon-juniper cover class 1 (%; 75 m) | 0 | 0.02 | 0.02 | 0.08 | 0.08 | 0.03 |
|  | Pinyon-juniper cover class 1 (%; 167 m) | 0 | **0.49** | 0.04 | 0.08 | 0.09 | 0.04 |
|  | Pinyon-juniper cover class 1 (%; 260 m) | **1** | 0.02 | - | 0.08 | 0.12 | 0.29 |
|  | Pinyon-juniper cover class 1 (%; 370 m) | - | - | **0.38** | - | 0.11 | **0.4** |
|  | Pinyon-juniper cover class 1 (%; 439 m) | 0 | 0.46 | 0.1 | 0.08 | 0.1 | 0.04 |
|  | Pinyon-juniper cover class 1 (%; 1,451 m) | 0 | 0.04 | 0.29 | 0.09 | 0.1 | 0.04 |
|  | Distance to pinyon-juniper cover class 1 (exponential) | 0 | 0 | 0.04 | 0.14 | 0.14 | 0.06 |
|  | Distance to pinyon-juniper cover class 2 (exponential) | 0 | 0 | 0.04 | **0.28** | **0.14** | 0.05 |
|  | Distance to all forest (exponential) | 0 | 0 | 0.1 | 0.17 | 0.13 | 0.06 |
| Burned area | Cumulative burned area (%; 260 m) | **0.45** | **0.51** | 0.17 | 0.27 | 0.25 | 0.27 |
|  | Cumulative burned area (%; 370 m) | - | - | 0.2 | - | 0.29 | **0.27** |
|  | Cumulative burned area (%; 439 m) | 0.38 | 0.33 | 0.22 | 0.23 | **0.3** | 0.26 |
|  | Cumulative burned area (%; 1,451 m) | 0.17 | 0.15 | **0.41** | **0.51** | 0.16 | 0.2 |
| Streams | Total stream density (km/km^2^; 75 m) | 0.02 | 0.15 | 0.01 | 0 | 0.04 | 0.05 |
|  | Total stream density (km/km^2^; 167 m) | 0.05 | 0.02 | 0.02 | 0.08 | 0.06 | 0.06 |
|  | Total stream density (km/km^2^; 260 m) | 0.03 | 0.02 | - | 0.05 | 0.06 | 0.04 |
|  | Total stream density (km/km^2^; 370 m) | - | - | 0.03 | - | 0.06 | 0.04 |
|  | Total stream density (km/km^2^; 439 m) | 0.03 | 0.02 | 0.04 | 0.06 | 0.07 | 0.03 |
|  | Total stream density (km/km^2^; 1,451 m) | 0.02 | 0.07 | 0.14 | 0.05 | 0.04 | 0.04 |
|  | Distance to any stream (exponential) | 0.0003 | 0.02 | 0.07 | 0.1 | 0.07 | 0.04 |
|  | Intermittent stream density (km/km^2^; 75 m) | 0.002 | 0.02 | 0.02 | 0 | 0.04 | 0.04 |
|  | Intermittent stream density (km/km^2^; 167 m) | 0.001 | 0.01 | 0.02 | 0.07 | 0.03 | 0.04 |
|  | Intermittent stream density (km/km^2^; 260 m) | 0.0003 | 0.01 | - | 0.05 | 0.03 | 0.04 |
|  | Intermittent stream density (km/km^2^; 370 m) | - | - | 0.05 | - | 0.03 | 0.03 |
|  | Intermittent stream density (km/km^2^; 439 m) | 0.005 | 0.01 | 0.05 | 0.06 | 0.03 | 0.04 |
|  | Intermittent stream density (km/km^2^; 1,451 m) | 0.009 | 0.02 | 0.03 | 0.05 | 0.04 | **0.18** |
|  | Distance to intermittent stream (exponential) | 0.004 | 0.02 | 0.06 | **0.11** | 0.05 | 0.05 |
|  | Perennial stream density (km/km^2^; 75 m) | 0.009 | 0.13 | 0.01 | 0 | 0.04 | 0.05 |
|  | Perennial stream density (km/km^2^; 167 m) | 0.06 | 0.04 | 0.02 | 0.06 | 0.05 | 0.05 |
|  | Perennial stream density (km/km^2^; 260 m) | 0.18 | 0.03 | - | 0.05 | 0.06 | 0.04 |
|  | Perennial stream density (km/km^2^; 370 m) | - | - | 0.06 | - | 0.06 | 0.03 |
|  | Perennial stream density (km/km^2^; 439 m) | **0.51** | 0.04 | 0.06 | 0.06 | **0.08** | 0.03 |
|  | Perennial stream density (km/km^2^; 1,451 m) | 0.09 | **0.34** | **0.28** | 0.05 | 0.04 | 0.04 |
|  | Distance to perennial stream (exponential) | 0.001 | 0.03 | 0.03 | 0.09 | 0.04 | 0.05 |
| Springs | Spring density (spring/km^2^; 75 m) | 0.006 | 0.25 | 0.13 | 0 | 0.04 | 0.11 |
|  | Spring density (spring/km^2^; 167 m) | 0.003 | 0.14 | 0.1 | 0 | 0.05 | 0.14 |
|  | Spring density (spring/km^2^; 260 m) | 0.02 | 0.16 | - | 0.17 | 0.15 | 0.19 |
|  | Spring density (spring/km^2^; 370 m) | - | - | 0.1 | - | **0.39** | 0.12 |
|  | Spring density (spring/km^2^; 439 m) | 0.09 | 0.07 | 0.1 | **0.33** | 0.3 | 0.1 |
|  | Spring density (spring/km^2^; 1,451 m) | **0.88** | 0.08 | 0.16 | 0.19 | 0.03 | **0.21** |
|  | Distance to spring (exponential) | 0.002 | **0.29** | **0.4** | 0.31 | 0.04 | 0.14 |
| Elevation | Elevation (75 m) | 0.23 | **0.29** | 0.19 | 0.18 | 0.17 | 0.16 |
|  | Elevation (167 m) | **0.28** | 0.25 | 0.19 | 0.2 | **0.18** | 0.16 |
|  | Elevation (260 m) | 0.26 | 0.22 | - | 0.2 | 0.17 | 0.16 |
|  | Elevation (370 m) | - | - | **0.24** | - | 0.16 | 0.16 |
|  | Elevation (439 m) | 0.2 | 0.17 | 0.24 | 0.21 | 0.16 | 0.17 |
|  | Elevation (1,451 m) | 0.03 | 0.08 | 0.14 | **0.22** | 0.17 | **0.19** |
| Topography | Topographic roughness (75 m) | 0.07 | 0.18 | 0 | 0.05 | 0.07 | 0.08 |
|  | Topographic roughness (167 m) | 0.07 | 0.09 | 0 | 0.06 | 0.07 | 0.08 |
|  | Topographic roughness (260 m) | 0.08 | **0.5** | - | 0.07 | 0.07 | 0.07 |
|  | Topographic roughness (370 m) | - | - | 0.0002 | - | 0.06 | 0.06 |
|  | Topographic roughness (439 m) | 0.1 | 0.003 | 0 | **0.17** | 0.08 | 0.06 |
|  | Topographic roughness (1,451 m) | 0.08 | 0.0003 | 0 | 0.06 | **0.17** | 0.06 |
|  | Slope (75 m) | 0.09 | 0.1 | 0 | 0.07 | 0.08 | 0.08 |
|  | Slope (167 m) | 0.14 | 0.07 | 0.007 | 0.13 | 0.07 | 0.08 |
|  | Slope (260 m) | **0.15** | 0.04 | - | 0.149 | 0.08 | 0.09 |
|  | Slope (370 m) | - | - | 0.49 | - | 0.07 | 0.1 |
|  | Slope (439 m) | 0.14 | 0.01 | **0.5** | 0.15 | 0.08 | 0.09 |
|  | Slope (1,451 m) | 0.09 | 0.001 | 0 | 0.08 | 0.09 | **0.14** |
| Temperature/ moisture | Heat load index (75 m) | 0.003 | 0.02 | 0.09 | 0.07 | 0.03 | 0.05 |
|  | Heat load index (167 m) | 0.003 | 0.02 | 0.03 | 0.06 | 0.05 | 0.05 |
|  | Heat load index (260 m) | 0.003 | 0.03 | - | 0.06 | 0.09 | 0.05 |
|  | Heat load index (370 m) | - | - | 0.04 | - | 0.14 | 0.05 |
|  | Heat load index (439 m) | 0.004 | 0.1 | 0.04 | 0.07 | **0.16** | 0.05 |
|  | Heat load index (1,451 m) | 0.05 | **0.53** | 0.08 | 0.05 | 0.07 | 0.04 |
|  | Compound topographic index (75 m) | 0.006 | 0.02 | 0.01 | 0.06 | 0.05 | **0.13** |
|  | Compound topographic index (167 m) | 0.004 | 0.03 | 0.01 | 0.08 | 0.04 | 0.06 |
|  | Compound topographic index (260 m) | 0.004 | 0.03 | - | **0.095** | 0.03 | 0.06 |
|  | Compound topographic index (370 m) | - | - | 0.01 | **-** | 0.03 | 0.05 |
|  | Compound topographic index (439 m) | 0.003 | 0.03 | 0.01 | 0.05 | 0.03 | 0.06 |
|  | Compound topographic index (1,451 m) | 0.009 | 0.02 | 0.01 | 0.09 | 0.03 | 0.09 |
|  | Transformed aspect (75 m) | 0.007 | 0.01 | **0.29** | 0.06 | 0.02 | 0.05 |
|  | Transformed aspect (167 m) | 0.02 | 0.01 | 0.09 | 0.06 | 0.03 | 0.04 |
|  | Transformed aspect (260 m) | 0.02 | 0.02 | - | 0.06 | 0.05 | 0.04 |
|  | Transformed aspect (370 m) | - | - | 0.1 | - | 0.05 | 0.05 |
|  | Transformed aspect (439 m) | 0.04 | 0.03 | 0.08 | 0.06 | 0.06 | 0.04 |
|  | Transformed aspect (1,451 m) | **0.82** | 0.09 | 0.1 | 0.07 | 0.06 | 0.04 |

**
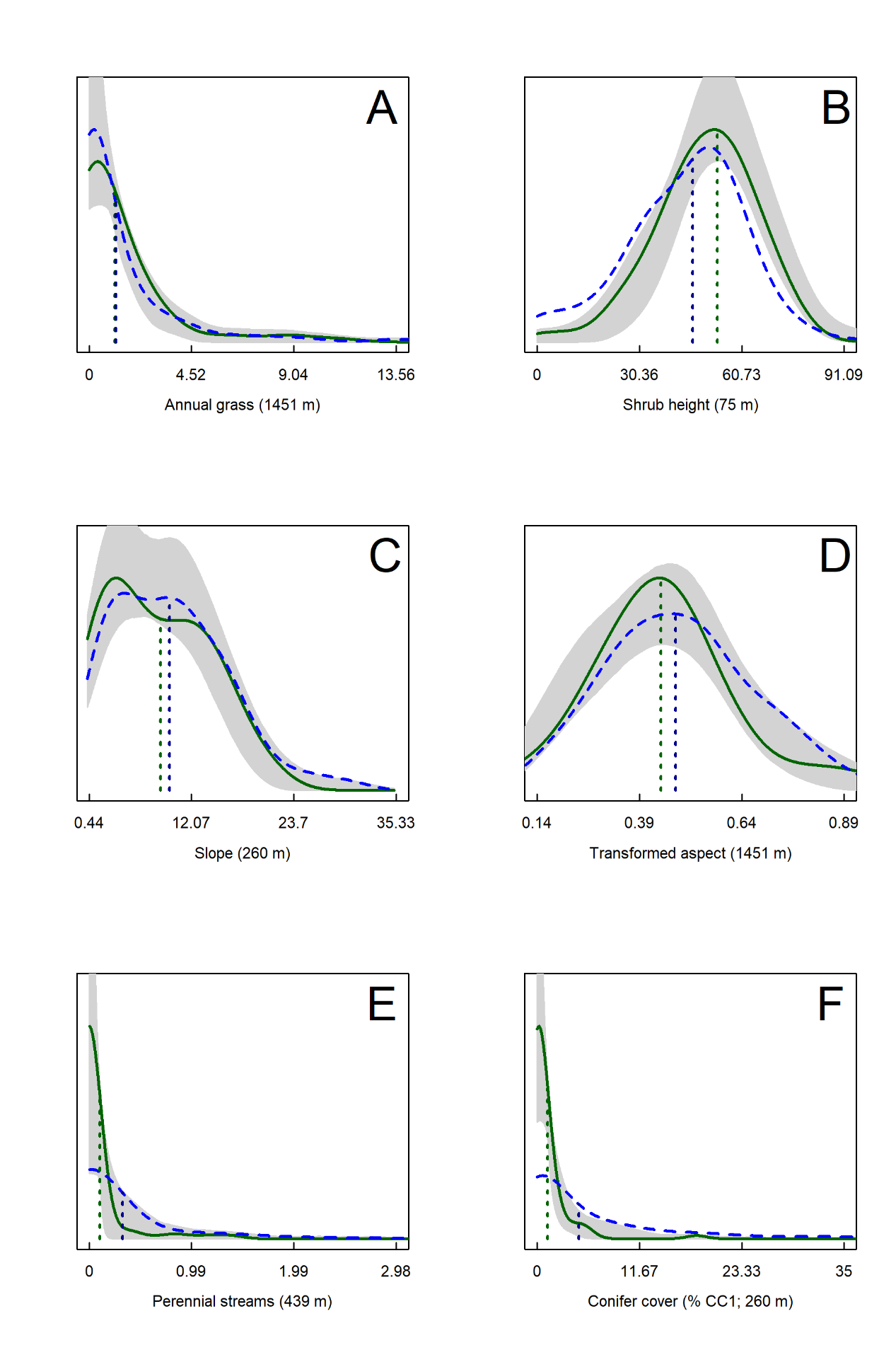

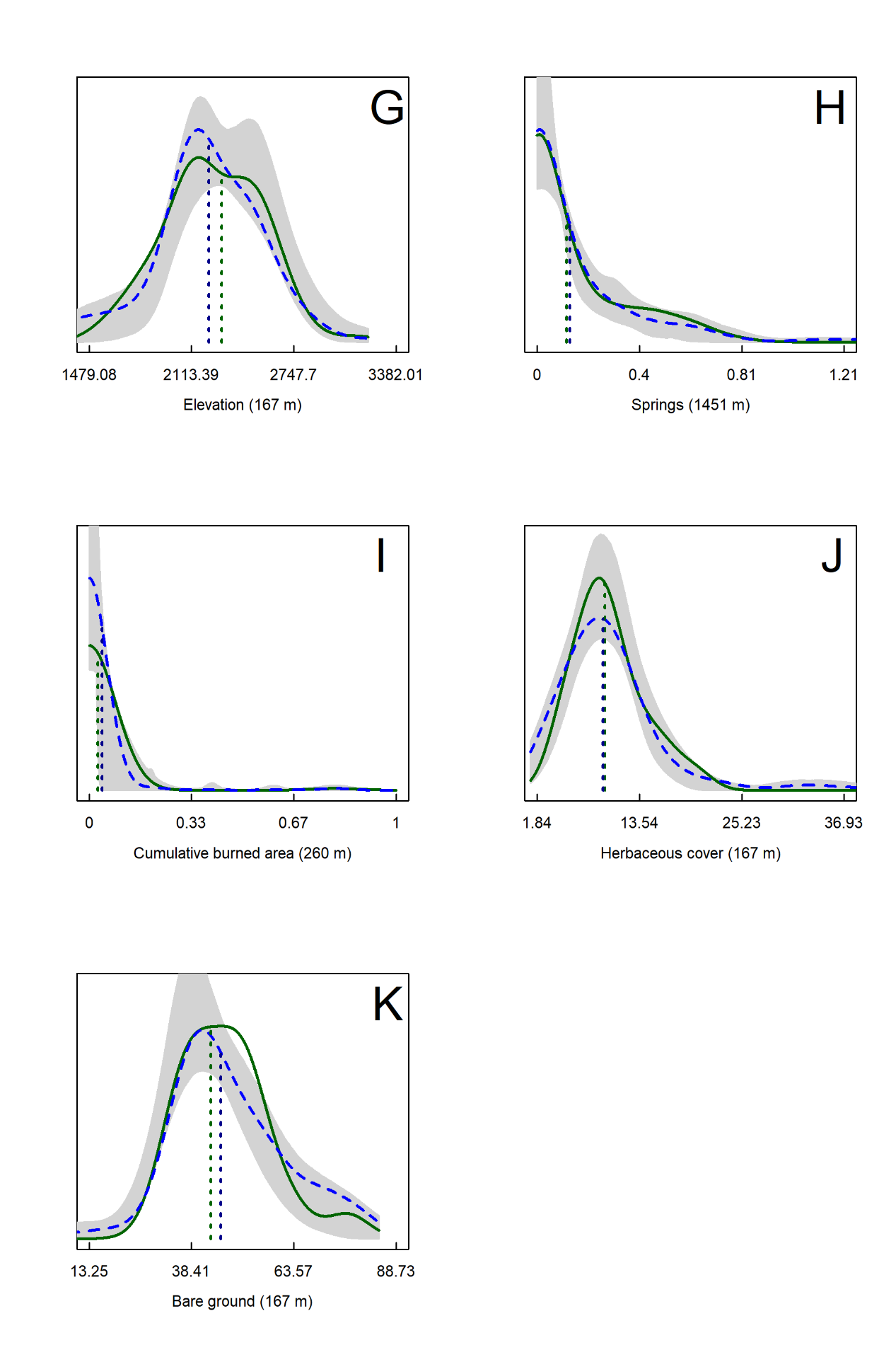
**

Figure S2. Used-habitat calibration plots for nest site selection analyses of greater sage-grouse in the Bi-State Distinct Population Segment from 2003–2019. Selection was evaluated for annual grasses (A), shrubs (B), slope (C), transformed aspect (D), streams (E), conifer cover (F), elevation (G), springs (H), burned area (I), herbaceous cover (J), and bare ground (K). A plot for saline lakes was not created due to data limitations within the independent testing dataset. The best spatial scale for each variable is noted parenthetically.

**
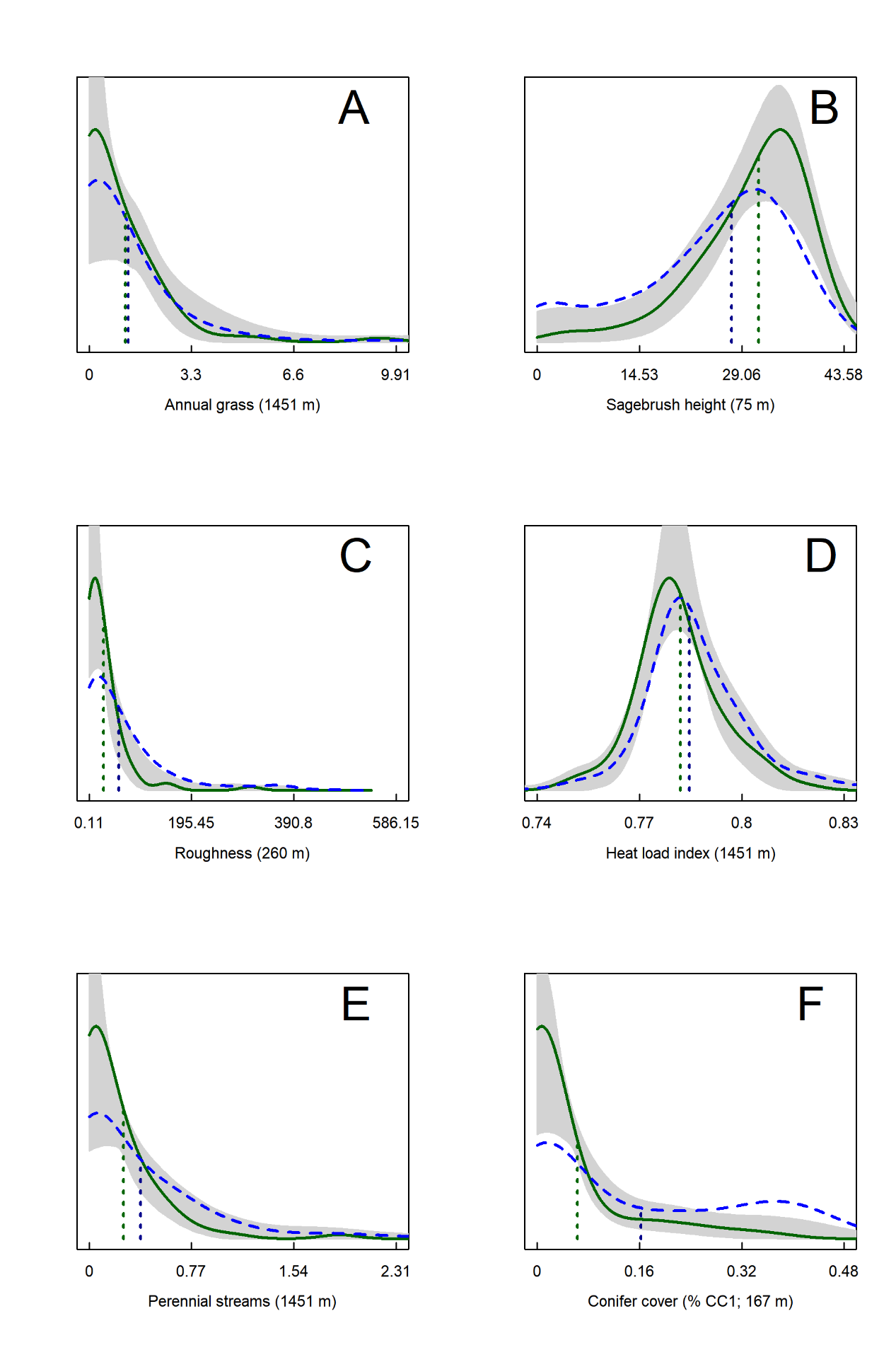

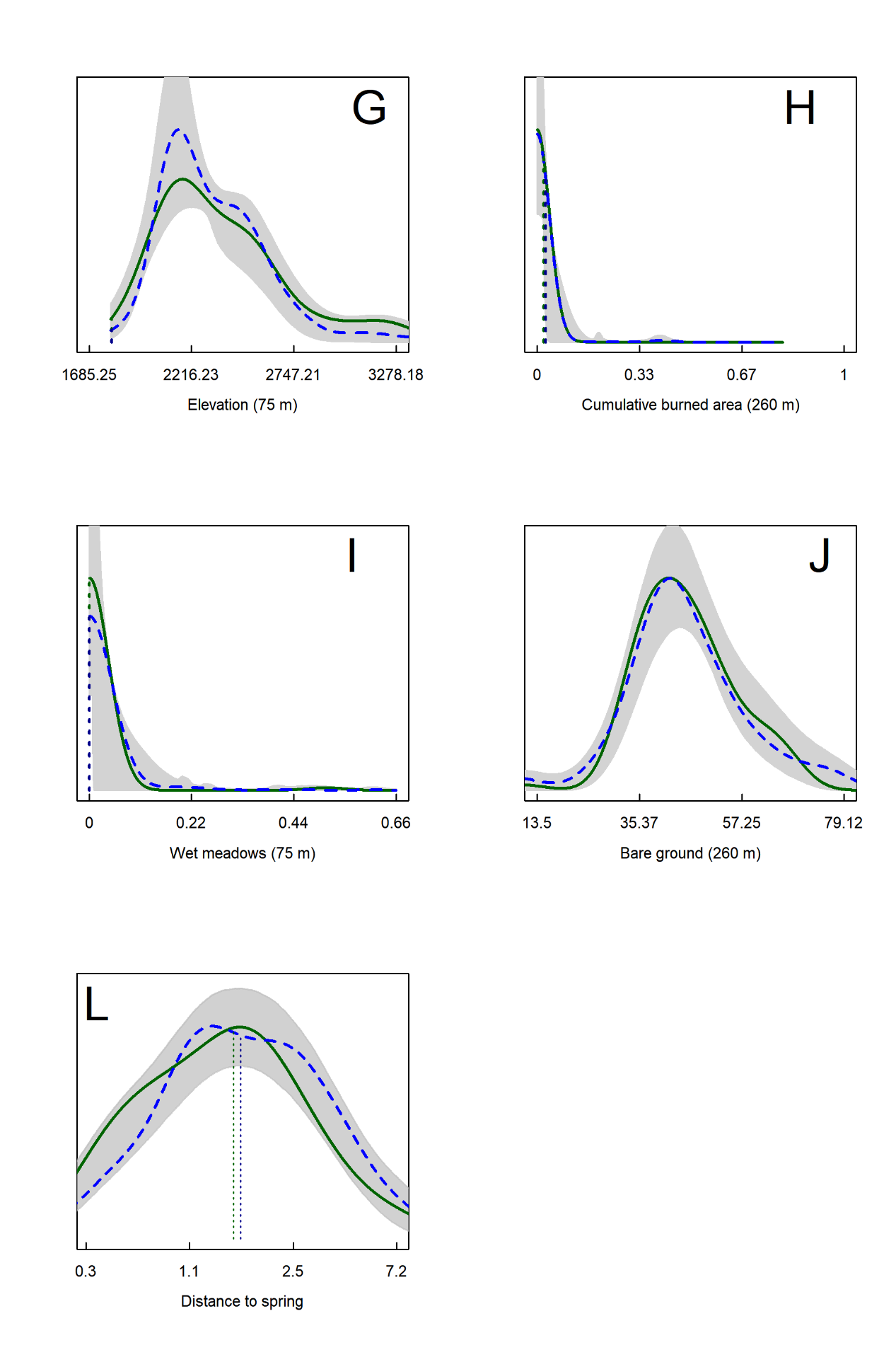
**

Figure S3. Used-habitat calibration plots for early brood selection analyses of greater sage-grouse in the Bi-State Distinct Population Segment from 2003–2019. Selection was evaluated for annual grasses (A), shrubs (B), roughness (C), heat load index (D), streams (E), conifer cover (F), elevation (G), burned area (H), wet meadows (I), bare ground (J), and springs (K). A plot for saline lakes was not created due to data limitations within the independent testing dataset. The best spatial scale for each variable is noted parenthetically.

**
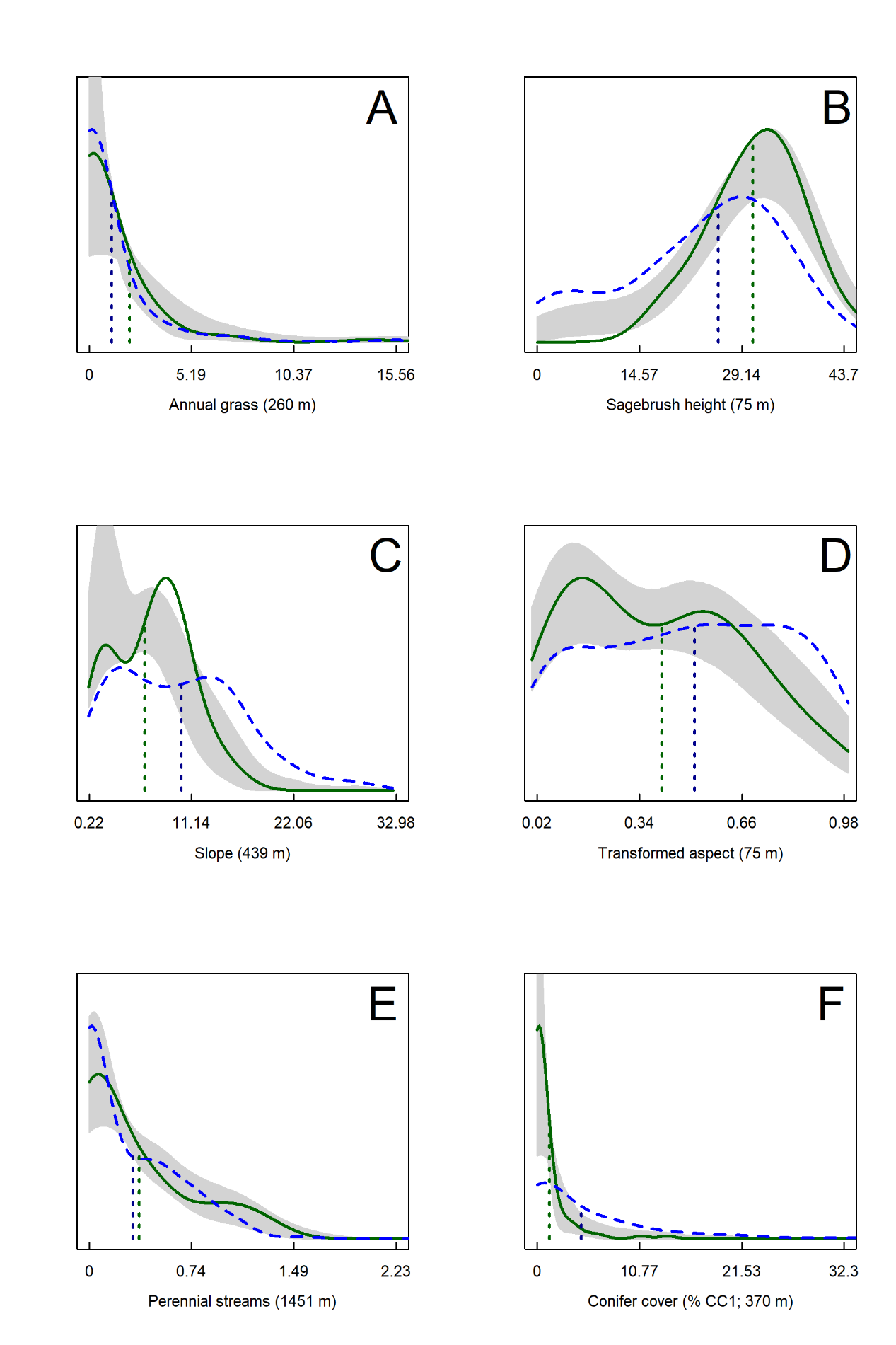

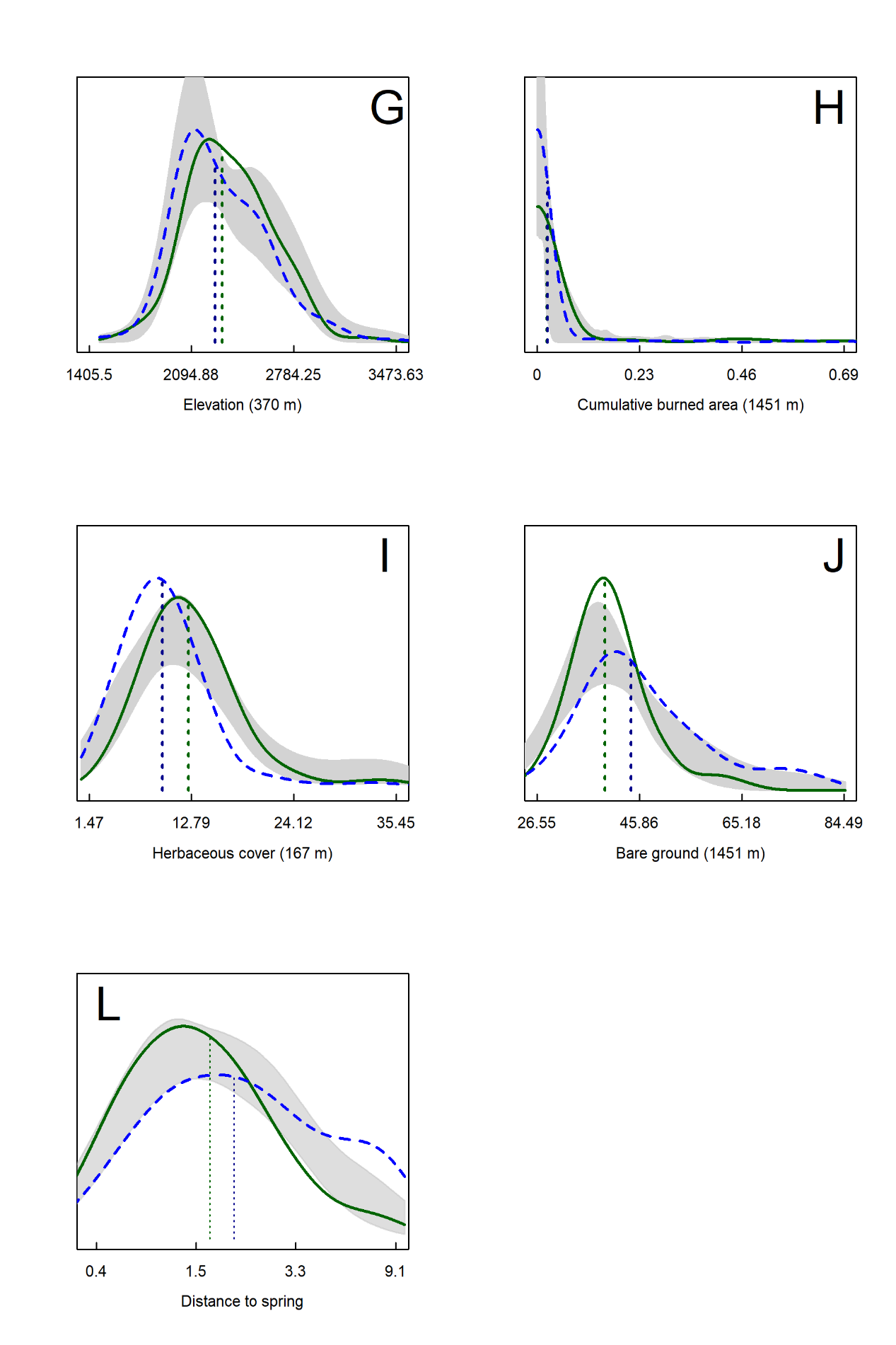
**

Figure S4. Used-habitat calibration plots for late brood selection analyses of greater sage-grouse in the Bi-State Distinct Population Segment from 2003–2019. Selection was evaluated for annual grasses (A), shrubs (B), slope (C), transformed aspect (D), streams (E), conifer cover (F), elevation (G), burned area (H), herbaceous cover (I), bare ground (J), and springs (K). A plot for saline lakes was not created due to data limitations within the independent testing dataset. The best spatial scale for each variable is noted parenthetically.
